# Evolution of the Bovine *TLR* Gene Family and Member Associations with *Mycobacterium avium* Subspecies *paratuberculosis* Infection

**DOI:** 10.1371/journal.pone.0027744

**Published:** 2011-11-30

**Authors:** Colleen A. Fisher, Eric K. Bhattarai, Jason B. Osterstock, Scot E. Dowd, Paul M. Seabury, Meenu Vikram, Robert H. Whitlock, Ynte H. Schukken, Robert D. Schnabel, Jeremy F. Taylor, James E. Womack, Christopher M. Seabury

**Affiliations:** 1 Department of Veterinary Pathobiology, College of Veterinary Medicine, Texas A&M University, College Station, Texas, United States of America; 2 Pfizer Animal Genetics, Kalamazoo, Michigan, United States of America; 3 Research and Testing Laboratory, SpiroStat Technologies, Medical Biofilm Research Institute, Lubbock, Texas, United States of America; 4 ElanTech, Inc., Greenbelt, Maryland, United States of America; 5 Department of Clinical Studies, School of Veterinary Medicine, University of Pennsylvania, Kennett Square, Pennsylvania, United States of America; 6 Department of Population Medicine and Diagnostic Sciences, College of Veterinary Medicine, Cornell University, Ithaca, New York, United States of America; 7 Division of Animal Sciences, University of Missouri, Columbia, Missouri, United States of America; Indian Institute of Science, India

## Abstract

Members of the Toll-like receptor (*TLR*) gene family occupy key roles in the mammalian innate immune system by functioning as sentries for the detection of invading pathogens, thereafter provoking host innate immune responses. We utilized a custom next-generation sequencing approach and allele-specific genotyping assays to detect and validate 280 biallelic variants across all 10 bovine *TLR* genes, including 71 nonsynonymous single nucleotide polymorphisms (SNPs) and one putative nonsense SNP. Bayesian haplotype reconstructions and median joining networks revealed haplotype sharing between *Bos taurus taurus* and *Bos taurus indicus* breeds at every locus, and specialized beef and dairy breeds could not be differentiated despite an average polymorphism density of 1 marker/158 bp. Collectively, 160 tagSNPs and two tag insertion-deletion mutations (indels) were sufficient to predict 100% of the variation at 280 variable sites for both *Bos* subspecies and their hybrids, whereas 118 tagSNPs and 1 tagIndel predictively captured 100% of the variation at 235 variable sites for *B. t. taurus*. Polyphen and SIFT analyses of amino acid (AA) replacements encoded by bovine *TLR* SNPs indicated that up to 32% of the AA substitutions were expected to impact protein function. Classical and newly developed tests of diversity provide strong support for balancing selection operating on *TLR3* and *TLR8*, and purifying selection acting on *TLR10*. An investigation of the persistence and continuity of linkage disequilibrium (r^2^≥0.50) between adjacent variable sites also supported the presence of selection acting on *TLR3* and *TLR8*. A case-control study employing validated variants from bovine *TLR* genes recognizing bacterial ligands revealed six SNPs potentially eliciting small effects on susceptibility to *Mycobacterium avium* spp *paratuberculosis* infection in dairy cattle. The results of this study will broadly impact domestic cattle research by providing the necessary foundation to explore several avenues of bovine translational genomics, and the potential for marker-assisted vaccination.

## Introduction

The ultimate goal of bovine genomics is the identification of genetic variation that modulates corresponding variation in economically important production traits, differential susceptibility to disease, and favorable host response to vaccines, which is expected to enable the improvement of these phenotypes via informed genomic selection (for review see [1]). The bovine genome sequence and first-generation HapMap projects [2], [3] have directly enabled genome-assisted selective breeding [1], nascent investigations of non-traditional traits such as marker-assisted vaccination (as diagnostics for enhanced vaccine design or animal response), the development of a new class of anti-infectives known as innate immunologicals [4], and the elucidation of loci that have evolved under strong selection, thus providing important computational evidence for genomic regions which may underlie economically important traits.

Relevant to the suppression of infectious diseases, the mammalian innate immune system provides host defense against a variety of pathogens without requiring prior exposure [5], [6]. Consequently, genes that modulate innate immunity have often been considered as candidate loci for improving host resistance to disease in agricultural species [7]-[10]. Among mammals, the Toll-like receptor genes (*TLR*s) facilitate host recognition of pathogen-associated molecular patterns (PAMPs), thereafter eliciting host innate immune responses [5], [6] aimed at suppressing invading bacteria, viruses, protozoa, and fungi. Essential to their role in host defense, the mammalian *TLR*s encode type I transmembrane proteins of the Interleukin-1 receptor (IL-1R) family with N-terminal leucine-rich repeats (LRR) involved in ligand recognition, a transmembrane domain, and a C-terminal intracellular Toll/IL-1 receptor homologous (TIR/IL-1R) domain for signal transduction [5], [6], [11]. The mammalian *TLR* genes are primarily expressed by antigen-presenting cells (i.e., macrophages or dendritic cells), and most of the *TLR* ligand specificities have been experimentally elucidated, with six gene family members (*TLR1*, *TLR2*, *TLR4*, *TLR5*, *TLR6*, *TLR9*) known to recognize microbial (bacteria, fungi, protozoa) and/or synthetic ligands, and five (*TLR3*, *TLR4*, *TLR7*-*TLR9*) known to recognize viral components [11], [12]. Presently, *TLR10* remains the only functional human *TLR* gene family member for which natural and/or synthetic ligands have not been fully elucidated [13]. However, given evidence for functional mammalian TLR protein heterodimers (TLR10/TLR1; TLR2/TLR10) [13], the host protein encoded by *TLR10* may collaboratively enable recognition of a diverse array of microbial PAMPs, including those recognized by TLR2 [13]-[16].

Several studies have demonstrated that some naturally occurring *TLR* variants enhance the risk of severe infections in humans, mice, and domestic cattle, including the potential for increased susceptibility to Johne's disease, a debilitating and economically important disease of ruminants caused by infection with *Mycobacterium avium* spp *paratuberculosis* (MAP) (for review see [17]-[22]). Furthermore, several important bovine health-related QTL have also been localized to genomic regions either proximal to or directly overlapping one or more *TLR* loci (for review see [8], [23]-[27]). Therefore, we utilized massively parallel pyrosequencing of a pooled *TLR* amplicon library (*TLR*s 1-10) to comprehensively evaluate nucleotide variation and haplotype structure for 31 cattle breeds representing *Bos taurus taurus*, *Bos taurus indicus,* and their subspecific hybrids (composites). Overall, 276 single nucleotide polymorphisms (SNPs) and 4 insertion-deletion (indel) mutations were detected and validated. Bovine *TLR* SNPs and indels leveraged from the pyrosequencing study were used in a case-control analysis to identify risk factors underlying differential susceptibility to MAP in U.S. dairy cattle. In addition, we also comprehensively report on bovine *TLR* haplotype structure, the extent of haplotype sharing among specialized breeds and subspecific lineages, and provide median joining networks as putative representations of bovine *TLR* haplotype evolution [28]. Finally, we provide computational evidence for several bovine *TLR* genes evolving under disparate modes of non-neutral evolution, thereby underscoring their potential importance to bovine innate immunity and health-related traits. The results of this study will enable bovine translational genomics, QTL refinement, and ultimately, genome-assisted methods for animal selection to develop cattle populations with enhanced disease resistance and favorable vaccine response.

## Results

### Bovine *TLR* pyrosequencing, SNP detection, variant validation, and haplotype inference

For 96 elite bovine sires representing 31 domestic cattle breeds (*B. t. taurus*; *B. t. indicus*; and composites), we generated and purified 81 amplicons targeting all 10 bovine *TLR* genes (n = 7,776 total amplicon targets; see methods). The majority of the amplicons were pooled (n = 6,816) to form a normalized fragment library (Table S1) which was subjected to a workflow involving Roche 454 Titanium pyrosequencing with downstream variant detection using the Neighborhood Quality Standard algorithm as recently described [29], and the remaining purified amplicons (n = 960) were analyzed by standard dye-terminator cycle sequencing (Sanger) with alignment-based variant detection [23]-[25]. Sanger sequencing was necessary for amplicons that were intolerant to the addition of 5′ oligonucleotide barcodes for PCR amplification. In total, 474 variable sites were predicted from intragenic analyses of all sequence data, which included 212 previously validated SNPs [30], 4 known insertion-deletion mutations (indels) [30], and 258 new putative SNPs. Evaluation of the genic distributions of all newly predicted *TLR* variable sites detected within the pyrosequencing data revealed that≥62% of the 258 new putative SNPs were located either within or immediately flanking homopolymer repeats. Nevertheless, to allow for inclusion of all possible SNPs in downstream analyses, we investigated all 474 variable sites via fluorescent allele-specific genotyping assays [30]. Collectively, we validated 280 biallelic *TLR* variants (276 SNPs + 4 indels; Table S2) using custom genotyping assays applied to the sequencing discovery panel (n = 96 elite sires; 31 breeds), a panel of Holstein dairy cattle (n = 405; 3 herds), and a panel of purebred Angus beef cattle from a single herd (n = 48).

**Figure 1:**
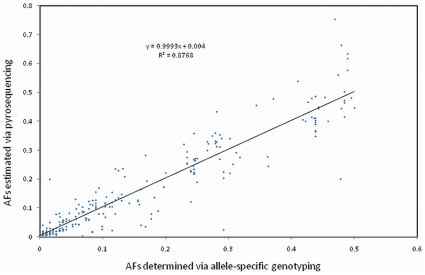
For validated bovine *TLR* SNPs detected via pyrosequencing (n = 244), a regression analysis was performed for pyrosequencing allele frequency (AF) estimates corresponding to the true minor alleles. (<0.5), as defined by allele-specific genotyping assays, and minor AFs (MAFs) directly ascertained by genotyping (n = 96 elite sires; 31 breeds). The true minor alleles (<0.5) were correctly identified for 236/244 (97%) SNPs via pyrosequencing. This analysis provided strong statistical evidence (*P* = 0.999846; Ho: slope = 1) for little or no bias in the pyrosequencing-based estimates of allele frequency.

Of the 276 validated SNPs, 71 were predicted to encode nonsynonymous substitutions (nsSNPs), and one was predicted to encode a nonsense mutation in bovine *TLR5* (AA substitution R125*; SNP C2332T). For the validated SNPs detected via pyrosequencing (n = 244), we investigated the relationship between minor allele frequencies (MAFs) estimated from the analysis of pyrosequencing data, as compared to corresponding allele frequencies derived from individual fluorescent allele-specific genotyping assays, and found significant correlations across all 10 *TLR* genes (discovery panel; Table 1). Moreover, an analysis performed across all genes (n = 244 SNPs) revealed that there was little or no bias in the estimates of allele frequencies produced via targeted pyrosequencing (*P* = 0.999846; Ho: slope = 1; Figure 1).

**Table 1:** Relationship between minor allele frequencies estimated from pyrosequencing and allele-specific genotyping of 96 individuals from 31 breeds.

Bovine Gene	Total 454 SNPs^a^	Overall Correlation (r)^b^	Overall RSQ (r^2^)^c^
*TLR1*	4	0.998	0.996
*TLR2*	44	0.935	0.874
*TLR3*	39	0.958	0.918
*TLR4*	28	0.948	0.898
*TLR5*	39	0.942	0.887
*TLR6*	15	0.879	0.773
*TLR7*	15	0.959	0.920
*TLR8*	13	0.877	0.769
*TLR9*	22	0.975	0.950
*TLR10*	25	0.749	0.561
Totals/Avg	244	0.922	0.855

a Total SNPs detected via pyrosequencing.

b *P*<0.05 for all *TLR* genes.

c RSQ is the squared correlation coefficient (r^2^).

Collectively, 266 SNPs and 4 indels were successfully incorporated into 243 unique haplotypes via Bayesian reconstructions [30], [31] (Table 2), which included one discrete haplotype carrying the putative *TLR5* nonsense SNP. Ten SNPs (*TLR2*: 9431, 10047, 12121; *TLR3*: 3624, 3804, 5201, 6382; *TLR4*: 8166; *TLR5*: 1562, 1685; see Table S2) could not be incorporated into discrete haplotypes with best-pair phase probabilities≥0.90. Summary data representing the total number of predicted haplotypes, number of cattle with phase probabilities≥0.90, total number of variable sites with MAF≤0.10, genic distributions of validated variable sites, size of the investigated regions, and average estimates of linkage disequilibrium (LD; r^2^) between adjacent variable sites are depicted in Table 2. Across all investigated loci (n = 549 cattle; 31 breeds), the MAF spectrum derived from allele-specific genotyping assays ranged from 0.001 to 0.498, with 64% of the validated SNPs possessing MAFs≤0.10 (Table 2).

**Table 2:** Summary data for validated polymorphisms detected in the bovine *TLR* gene family.

BovineGene	BTA Assign^a^	TotalHaps^b^	Sires Phased^c^	MAF≤0.10^d^	Avg r^2^ all^e^	Avg r^2^ *B.t.t.* e	Valid.SNPs^f^	HapSNPs^g^	Valid.Indels^h^	Valid.nsSNPs^i^	RegionSize^j^ (Kb)	QTL orAssoc.^k^
*TLR1*	BTA6	8	547	3	0.24	0.49	5	5	0	2	2.184	Q
*TLR2*	BTA17	38	532	38	0.19	0.24	44	41	1	20	3.224	Q, A^l^
*TLR3*	BTA27	40	78	20	0.29	0.57	56	52	0	3	9.469	A
*TLR4*	BTA8	29	532	23	0.10	0.08	28	27	0	7	3.470	Q, A
*TLR5*	BTA16	29	526	29	0.20	0.31	43	41	3	9	5.334	No
*TLR6*	BTA6	20	526	13	0.09	0.12	15	15	0	6	2.327	Q, A^l^
*TLR7*	BTAX	9	96	7	0.28	0.28	15	15	0	1	4.285	Q
*TLR8*	BTAX	6	96	1	0.70	0.69	13	13	0	8	3.702	Q
*TLR9*	BTA22	20	545	9	0.27	0.29	22	22	0	3	5.033	Q
*TLR10*	BTA6	43	524	34	0.27	0.15	35	35	0	13	3.859	Q^l^
**Total/Avg**		**243**	**96%**	**177**	**0.26**	**0.32**	**276**	**266**	**4**	**72**	**42.887**	

a BTA assignments based on NCBI Refseq (Btau5.2).

b Total number of haplotypes predicted from all validated markers and best pair reconstructions [31] with probabilities≥0.90.

c Number of cattle exhibiting best pair phase probabilities≥0.90. BTAX haplotypes were directly ascertained. 96 animals were genotyped for *TLR3*, *TLR7*, and *TLR8*. For all other loci, 549 animals were genotyped.

d Number of polymorphisms with minor allele frequencies≤0.10.

e Average intragenic linkage disequilibrium (r^2^) values estimated for adjacent SNP and indel sites for all cattle or for *B. t. taurus* (*B.t.t.*).

f Number of putative SNPs validated as polymorphic.

g Number of validated SNPs incorporated into discrete haplotypes.

h Number of putative indels validated as polymorphic.

i Number of nonsynonymous SNPs validated as polymorphic, including the putative *TLR5* nonsense SNP.

j Size of the genic region. Kb = Kilobase.

k Bovine health-related QTL overlapping or proximal to investigated gene (Q), or intragenic variation associated (A) with disease susceptibility in case-control studies [19]-[27], [46].

l Tentative association in this study.

### Characterization of LD architecture, recombination, and intragenic tagSNPs/Indels

Evaluation of the intragenic patterns of LD across all 31 breeds of cattle via 95% confidence intervals constructed for D' [32], [33], application of the four gamete rule [32], and estimates of recombination between adjacent variable sites [34], [35] revealed one or more blocks of strong LD within each of the 10 bovine *TLR* genes. Statistical evidence for historical recombination was detected within *TLR2*, *TLR3*, and *TLR6*, resulting in at least two detectable LD blocks within each gene. All other genes exhibited a single block of strong LD spanning either all, or the majority of all validated intragenic SNPs and indels, as supported by the majority rule of all three analyses [32]-[35]. A comparison of average intragenic r^2^ values calculated between adjacent variable sites across all 10 genes revealed a dynamic range of LD (0.09-0.70; all cattle, 31 breeds; Table 2). Discrete regions of high and low LD, the latter due to historical recombination, were also detected using the general model for varying recombination rate [31], [34], [35]. Cumulatively, four adjacent SNP sites [*TLR2* (1), *TLR3* (2), and *TLR6* (1)] produced estimates of median recombination rates that exceeded the background rate () [31], [34], [35] by a factor of at least 2.5. The highest median estimate of recombination rate was observed in *TLR3* (between SNP positions rs42851925, rs55617222; rs55617241, rs55617451, Table S2), and exceeded the background rate by a factor of at least 5.2. Analyses to identify tagSNPs/Indels which predictively captured 100% of the variation at 280 validated variable sites within all 10 genes for all cattle yielded 160 tagSNPs and 2 tagIndels (Table S3). Similar analyses restricted to the *B. t. taurus* breeds demonstrated that only 118 tagSNPs and 1 tagIndel were predicted to capture 100% of the variation at 235 variable sites (Table S3). Interestingly, the cumulative tagging efficiency (total tags predicted/total number of validated variable sites) was similar for both analyses (all cattle vs *B. t. taurus*), with this result largely due to the preponderance of taurine cattle in the total sample (94.4%), and the significant sharing of SNPs, indels, and haplotypes among the subspecific lineages.

### High resolution bovine *TLR* haplotype networks and breed distributions

**Figure 2:**
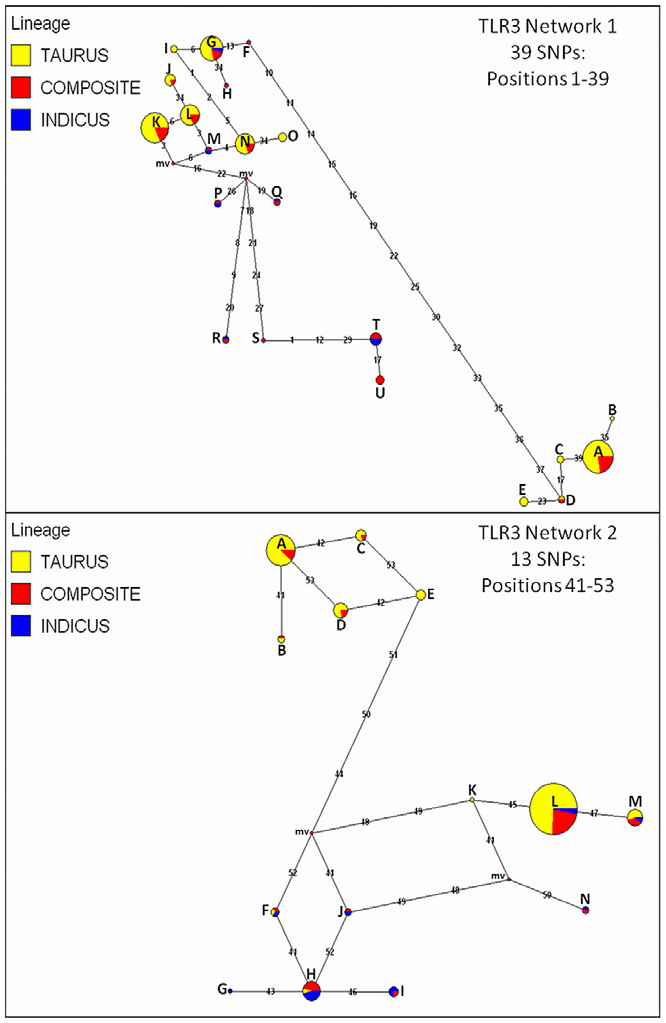
Median joining (MJ) haplotype networks for bovine *TLR3* using haplotypes predicted for all cattle (n = 96 AI sires, 31 breeds). Because MJ networks require the absence of recombination [66], each network represents intragenic regions of elevated LD. Haplotypes predicted for *B. t. taurus*, *B. t. indicus* and hybrids (termed “composites”) are color coded. Numbers indicate SNP positions in numerical order (see Table S2 for SNP information). Node sizes are proportional to haplotype frequency, and all branch lengths are drawn to scale. Alphabetized letters at nodes represent the breed distribution of each haplotype (Table S4). Median vectors are indicated as “mv”.

**Figure 3:**
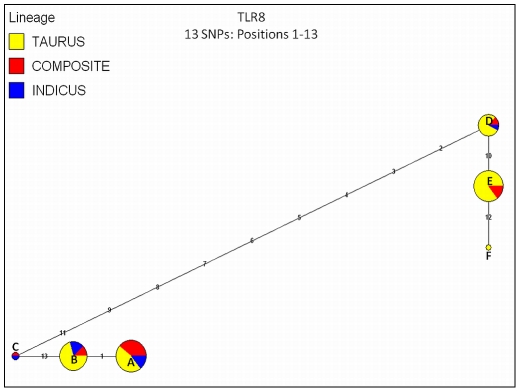
Median joining (MJ) haplotype network for bovine *TLR8* using haplotypes directly ascertained for all cattle (n = 96 AI sires, 31 breeds). Haplotypes observed for *B. t. taurus*, *B. t. indicus* and hybrids (termed “composites”) are color coded. Numbers indicate SNP positions in numerical order (see Table S2 for SNP information). Node sizes are proportional to haplotype frequency, and all branch lengths are drawn to scale. Alphabetized letters at nodes represent the breed distribution of each haplotype (Table S4).

**Figure 4:**
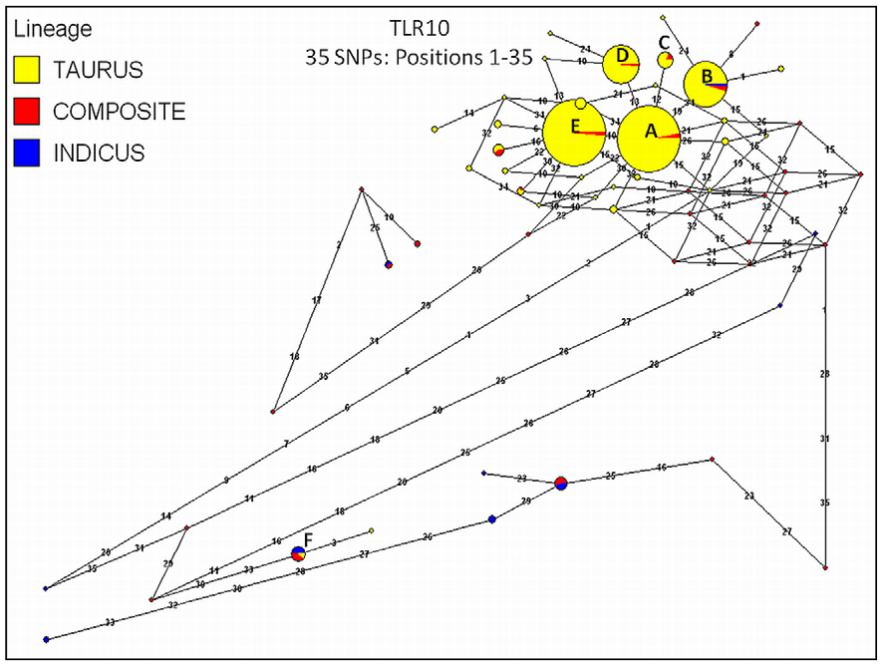
Median joining (MJ) haplotype network for bovine *TLR10* using haplotypes predicted for all cattle (n = 96 AI sires, 31 breeds; 48 Purebred Angus; 405 Holstein cattle). Haplotypes predicted for *B. t. taurus*, *B. t. indicus* and hybrids (termed “composites”) are color coded. Numbers indicate SNP positions in numerical order (see Table S2 for SNP information). Node sizes are proportional to haplotype frequency, and all branch lengths are drawn to scale. Alphabetized letters at nodes represent the breed distribution of each haplotype (Table S4). Notably, given the complexity of the network, only nodes representing≥10 cattle are labeled (A-F), which collectively represents>93% of the cattle meeting the phase requirements (n = 524 cattle with best-pair probabilities≥0.90). Median vectors are indicated as “mv”.

Median joining haplotype networks (Figures 2,3,4, Figure S1; Table S4) constructed for all 10 genes revealed that: 1) The specialized *B. t. taurus* beef and dairy breeds cannot be genetically discriminated despite an average polymorphism density (266 SNPs + 4 indels; see Table 2) of one variable marker per 158 bp; 2) Haplotype sharing occurs among *B. t. taurus* and *B. t. indicus* breeds at all 10 loci; 3) Shared haplotypes were often the highest frequency haplotype(s) within a network; 4) Despite haplotype sharing between the subspecific lineages, the 250 Kyr divergence [36] between *B. t. taurus* and *B. t. indicus* was evident in most, but not all, haplotype networks (i.e., *TLR1-7, TLR10*). With very few exceptions (i.e., *TLR3* Network 1, *TLR4*, *TLR10*), the high frequency network nodes demonstrating subspecific haplotype sharing often included at least two indicine sires. Using summary data derived from the median joining networks (Table S4), we estimated the relationship between the total number of discrete *TLR* haplotypes predicted (*TLR1-10*) in seven major U.S. taurine beef breeds [37] (Angus, Charolais, Gelbvieh, Hereford, Limousin, Red Angus, Simmental), and four U.S. taurine dairy breeds (Braunvieh, Brown Swiss, Holstein, Shorthorn), and found a significant correlation (r = 0.71, *P*≤0.0224). This correlation was driven by the large number of haplotypes predicted to be shared among the beef and dairy breeds. For the investigated beef breeds, we predicted 84 discrete haplotypes across all 10 *TLR* loci, and at least 60 (71.4%) were predicted to be shared with the four dairy breeds. However, we also detected disparities between the numbers of haplotypes predicted for *TLR4* and *TLR5*, with the dairy breeds possessing 3.8X and 2.3X more discrete haplotypes for these loci, respectively, than did our beef cattle. Exclusion of these two outlying loci resulted in a nearly perfect correlation (r = 0.98, *P*<0.0001) between the numbers of discrete haplotypes predicted in beef and dairy breeds across the remaining *TLR* loci. Interestingly, the single haplotype possessing the *TLR5* putative nonsense mutation was almost exclusively predicted in Holstein cattle (Figure S1, *TLR5* Node Q; n = 53 Holstein, n = 1 Braford).

### Functional modeling of bovine amino acid (AA) substitutions and tests of selection

Using both PolyPhen [38] and SIFT [39] to evaluate the putative functional effects of AA substitutions encoded by *TLR* SNPs, we determined that 54/72 (75%) of AA substitutions were predicted to be benign and tolerated, whereas 23/72 (32%) were predicted to impact protein function [40] by at least one of the analytical methods employed (Table 3). For those mutations predicted to impact protein function, 18/23 (78%) were detected at frequencies<0.05, and 5/23 (22%) located in *TLR2* (1), *TLR3* (2), *TLR5* (1; putative nonsense SNP), and *TLR8* (1) were observed at frequencies≥0.05, with moderate frequency substitutions detected in *TLR8* (0.562) and *TLR3* (0.432; see Table 3). The MAF for the *TLR5* putative nonsense SNP, as estimated from 405 Holsteins in three herds was 0.068 (Table 3). Across all polymorphisms involving AA substitutions, PolyPhen and SIFT produced analogous predictions for 61/72 (85%) observed replacements.

**Table 3:** Summary data for 22 nonsynonymous SNPs and one putative nonsense SNP predicted to impact protein function.

Bovine Gene	SNP^a^	dbSNP ID	GenBank Protein ID	AA Subst.^b^	Protein Domain^c^	PolyPhen Result^d^	SIFT Result^d^	SNP Freq^e^
*TLR2*	G>T	*ss470256478*	NP_776622.1	W119L	LRR_TYP1	PrD	AF	0.008
	T>A	*rs68268251*	NP_776622.1	F227L	NCP	PsD	T	0.015
	C>T	*ss470256481*	NP_776622.1	T311M	NCP	PrD	AF	0.006
	C>T	*ss470256483*	NP_776622.1	S485F	LRR_TYP2	PrD	AF	0.015
	G>A	*rs68268260*	NP_776622.1	R563H	LRRCT	B	AF	0.066
	G>C	*ss470256484*	NP_776622.1	E738Q	TIR	PsD	AF	0.001
*TLR3*	G>A	*rs55617272*	NP_001008664.1	G426S	LRR8	PsD	AF	0.058
	G>T	*rs42852439*	NP_001008664.1	S664I	LRRCT	PsD	T	0.432
*TLR4*	A>C	*rs8193049*	NP_776623.5	N151T	LRR3	PsD	T	0.009
	A>G	*rs8193055*	NP_776623.5	K381R	LRR6	B	AF	0.005
	A>G	*ss469376075*	NP_776623.5	H587R	LRRCT	PrD	AF	0.003
*TLR5*	C>T	*ss469376099*	NP_001035591.1	R125*	NCP	PsD	ND	0.053^f^
	G>A	*ss469376101*	NP_001035591.1	R262H	NCP	PrD	T	0.004
	C>G	*ss469376107*	NP_001035591.1	F643L	NCP	B	AF	0.003
*TLR6*	T>G	*rs68268270*	NP_001001159.1	L43R	NCP	PrD	AF	0.003
	A>G	*rs68268272*	NP_001001159.1	R87G	LRR1	B	AF	0.017
	T>A	*ss469376113*	NP_001001159.1	F494I	LRR5	PrD	AF	0.024
*TLR7*	A>G	*ss469376123*	NP_001028933.1	N439S	NCP	PrD	AF	0.021
*TLR8*	G>A	*rs55617351*	ABQ52584.1	S477N	NCP	B	AF	0.562
	A>C	*ss469376137*	ABQ52584.1	K903T	TIR	PsD	AF	0.010
*TLR10*	G>A	*rs55617437*	NP_001070386.1	R18H	SigPep	PsD	T	0.018
	C>G	*rs55617286*	NP_001070386.1	I134M	LRR3	B	AF	0.013
	A>C	*rs55617297*	NP_001070386.1	K753T	TIR	PsD	AF	0.010

a SNPs with “rs” numbers were previously described [23]-[25], [30], [59] and validated in this study.

b Amino acid (AA) substitutions predicted from corresponding SNPs, GenBank Proteins, and previous studies [23]-[25], [30], [60].

c Protein domain locations predicted by SMART (http://smart.embl-heidelberg.de/). Only confidently predicted domains are depicted (NCP = no confident prediction; LRRs are named in order of prediction).

d Results from PolyPhen and SIFT [38]-[39]. Results other than “Benign (B)” or “Tolerated (T)” are predicted to be Possibly Damaging (PsD), Probably Damaging (PrD), or Affect Protein Function (AF). SIFT could not be used (ND) to model the *TLR5* putative nonsense SNP.

e Observed frequency of nonsynonymous SNP allele across all 31 cattle breeds.

f The frequency of this SNP in U.S. dairy cattle (n = 405, 3 Herds) was 0.068.

To collectively estimate the extent of functional and/or selective constraint(s) related to bovine TLR protein function, we used a goodness of fit test to examine disparities between the observed distributions of AA phenotypes (PolyPhen + SIFT results; benign/tolerated vs damaging/affect). Assuming equal probabilities for the occurrence of both classes of AA phenotypes across all bovine TLRs, we found there to be significantly fewer substitutions predicted to impact protein function than those classified as benign or tolerated (*P* = 0.00022). This is consistent with some degree of functional and/or selective constraints that generally operate to maintain the functional products of most protein coding genes [40]-[42]. However, this result describes a general trend across the bovine *TLR* gene family, and does not provide locus-specific insights regarding the evolutionary origin and magnitude of these constraints.

To elucidate gene-specific departures from a strictly neutral model of molecular evolution, we used Tajima's frequency distribution test (*D* statistic) [43], as applied to the discovery panel samples (all cattle from 31 breeds vs *B. t. taurus*), and evaluated the significance of the observed values (*D*) via coalescent simulation (Table 4). Departures from neutrality were detected for *TLR3*, *TLR8*, and *TLR10*. However, the direction of the deviation was not uniform across all three loci (Table 4), suggesting that disparate modes of evolution (i.e., selection) may have influenced genetic diversity within these genes, and that there may be differences among cattle lineages (Table 4, *TLR10*). For both *TLR3* and *TLR8*, a significantly positive Tajima's *D* reflected an excess of moderate frequency alleles, whereas a large negative value for *TLR10* (*B. t. taurus*) reflected an overabundance of rare, low frequency variants consistent with purifying selection [30]. Therefore, it is important to note that although a significant nonrandom trend toward benign or tolerated AA substitutions was detected across all investigated loci, the underlying reason for this functional and/or selective constraint appears to be fundamentally different between some gene family members (i.e., *TLR3*, *TLR8* vs *TLR10*). Notably, we observed at least one moderate frequency AA substitution that was predicted to impact protein function in both *TLR3* and *TLR8* (Table 3), whereas all AA substitutions predicted to impact protein function in *TLR10* were detected at very low frequencies (Table 3). To further investigate the overall magnitude and origin(s) of the most significant deviations from a strictly neutral model (Tajima's *D*; pyrosequencing discovery panel; Table 4), we used Fu's *F_S_* statistic [44] to estimate the probability of observing a number of haplotypes less than or equal to that predicted in our samples for *TLR3* (*B. t. taurus*); *TLR3-1* (*B. t. taurus*), and *TLR8* (all cattle; *B. t. taurus*). For *TLR3*, we recognized that the inability to phase all individuals in the pyrosequencing discovery panel could lead to the absence of some low frequency alleles, thus potentially driving both Tajima's *D* and Fu's *F_S_* toward larger positive values. Consequently, we calculated Fu's *F_S_* and Tajima's *D* for *TLR3* (*B. t. taurus*) and *TLR3*-1 (*B. t. taurus*) using the following approach: 1) Both test statistics were first calculated only for sires that could be phased with best-pairs probabilities≥0.90, as depicted in Table 4; and 2) If a significant result was achieved in this analysis, we then added the taurine haplotypes with phase probabilities<0.90 into our analyses (*D*; *F_S_*) by choosing the best haplotype pairs reconstructed for each sire. For Fu's *F_S_*, only *TLR8* displayed unequivocal evidence for a departure from neutrality (All cattle *F_S_* = 10.2712, *P*<0.01; *B. t. taurus F_S_* = 10.296, *P*<0.01), with levels of significance that withstood conservative correction for multiple testing (correction = α/n locus-specific tests, 0.05/2 = Minimal *P*≤0.025). For Tajima's *D*, inclusion of the best *TLR3* haplotype pairs for sires with phase probabilities<0.90 resulted in very similar test statistics (*TLR3 B. t. taurus D* = 3.6034, *P*<0.001; *TLR3-1 B. t. taurus D* = 3.4895, *P*<0.002; Table 4), with levels of significance that endured correction for multiple testing (0.05/8 = Minimal *P*≤0.00625).

**Table 4:** Summary data for tests of selection across all members of the bovine *TLR* gene family.

Gene	SiresPhased^a^	Tajima's*D* all^b^	Coalescent*P*-value^c^	Sires Phased^a^	Tajima's *D taurus* b	Coalescent*P*-value^c^
*TLR1*	95 (99%)	0.55535	*P*>0.05	64 (98%)	1.49328	*P*>0.05
*TLR2*	92 (96%)	0.51385	*P*>0.05	64 (98%)	-0.06547	*P*>0.05
*TLR3*	78 (81%)	2.35965	*P*<0.03	54 (83%)	3.63792	*P*<0.001e, f
*TLR3-1* d	83 (86%)	2.12744	*P*<0.04	59 (91%)	3.59176	*P*<0.001e, f
*TLR3-2* d	94 (98%)	2.07897	*P*<0.05	63 (97%)	2.65634	*P*<0.02
*TLR4*	89 (93%)	-0.83191	*P*>0.05	64 (98%)	0.93683	*P*>0.05
*TLR5*	86 (90%)	0.69344	*P*>0.05	59 (91%)	0.44166	*P*>0.05
*TLR6*	91 (95%)	0.16727	*P*>0.05	65 (100%)	-0.71248	*P*>0.05
*TLR7*	96 (100%)	-0.19828	*P*>0.05	65 (100%)	-0.17037	*P*>0.05
*TLR8*	96 (100%)	3.53957	*P*<0.001^e^	65 (100%)	3.28763	*P*<0.001^e^
*TLR9*	95 (99%)	1.15800	*P*>0.05	64 (98%)	1.26794	*P*>0.05
*TLR10*	92 (96%)	-0.29809	*P*>0.05	61 (94%)	-1.78285	*P*<0.03

a Number and proportion of cattle from the sequencing discovery panel with best-pair phase probabilities≥0.90 for all cattle (n = 96), and for *B. t. taurus* cattle (n = 65).

b Tajima's *D* statistic [43] for all cattle and for *B. t. taurus* breeds.

c Significance levels were estimated by coalescent simulation using 10,000 replicates [67]. All bolded loci were also significant (*P*<0.05) via application of the beta distribution [67].

d Phased variation within *TLR3* Network 1 and *TLR3* Network 2.

e Significant after correction for multiple tests (α / n locus-specific tests; α = 0.05).

f Significant after adding in the best-pairs of haplotypes for taurine sires with probabilities<0.90 and correction for multiple testing (α = 0.05).

**Figure 5:**
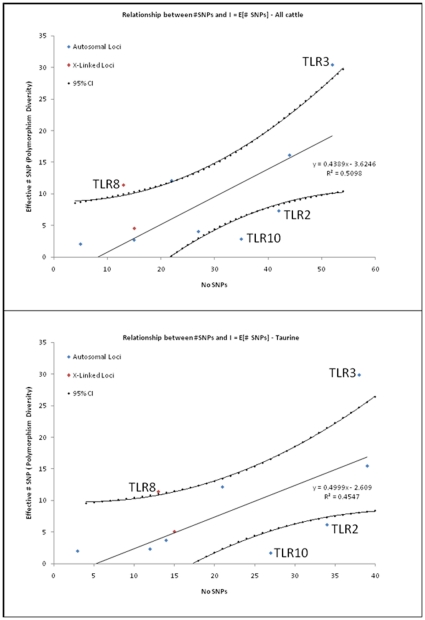
Relationship between the number of validated SNPs and SNP diversity here denoted as the effective number of SNPs across all 10 *TLR* loci in A) all cattle, and B) taurine cattle. The linear regressions and estimated 95% confidence intervals are shown in each panel.

A regression-based approach considering all validated variable sites and the effective number of SNPs at each site [30] also demonstrated that *TLR3* and *TLR8* possess significantly more gene diversity than do the eight other *TLR* loci (*P*≤0.05; Figure 5) in taurine and all cattle combined. In contrast, both regression analyses (all cattle; *B. t. taurus* only) indicated that *TLR10* and *TLR2* possess significantly less gene diversity than other members of the bovine *TLR* gene family (Figure 5). With the exception of *TLR2*, these results are precisely congruent with the results of Tajima's test (*D*; Table 4).

### Single Marker and Haplotype Association Tests with MAP Infection

Unphased diploid genotypes for a subset of the validated SNPs and indels (n = 35; nonsynonymous, putative nonsense, 5' upstream regions, and introns) within bovine *TLR* genes either known or postulated to primarily recognize bacterial ligands (*TLR1, TLR2, TLR4, TLR5, TLR6, TLR9, TLR10*) were tested for associations with bacterial culture status for MAP (fecal and/or tissue) in three Holstein dairy herds (n = 68 cases, 270 controls). All nonsynonymous *TLR* SNPs previously associated with MAP infection [19] (*TLR1*, *TLR2*, *TLR4*) were monomorphic in our samples (n = 549; 31 breeds). Conditional logistic regression models were constructed for each of 35 variable sites meeting our selection criteria (see methods) to estimate the relative odds of MAP infection given the defined diagnostic criteria adjusted for the effects of herd and age. Collectively, six SNPs produced suggestive associations, as evidenced by uncorrected *P*-values (Table 5). Interestingly, three SNPs in *TLR2* and one in *TLR6* were associated with increased odds of MAP infection in animals with 1 or more copies of the minor allele (Table 5). Two SNP loci, 1 in *TLR4* and 1 in *TLR10*, were associated with decreased odds of infection given increasing copies of the minor allele (Table 5). Following locus-specific correction of the *P*-values using the FDR method (http://sdmproject.com/utilities/? show = FDR) [45], two SNPs (*TLR6*-rs43702941; *TLR10*-rs55617325) remained significant (*P*≤0.05), and three SNPs (*TLR2*-rs68268245, ss470256479, rs43706433) displayed *P*-values (*P*≤0.053) that were suggestive of a potential recessive genetic association with MAP infection (Table 5). Two of these SNPs (*TLR2-*ss470256479, rs43706433) were recently hypothesized to occur on a haplotype associated with an increased risk for Johne's disease [46]. Consequently, we used PHASE 2.1 [31] to test the hypothesis that haplotype frequencies for bacterial-sensing *TLR*s differ between cases and controls. However, none of the investigated loci possessed significantly different haplotype distributions between cases and controls (*P>*0.05; 1,000 permutations).

**Table 5:** Summary statistics for single marker association tests with risk of *Mycobacterium avium* spp *paratuberculosis* infection.

					95% Confidence Interval^a^	
Marker	dbSNP ID	Model	Odds Ratio	*P*-value^c^	Lower Bound	Upper Bound
*TLR2-SNP 9564*	*rs68268245*	Recessive	3.20	0.032^d^	1.11	9.24
*TLR2-SNP 10511*	*ss470256479*	Recessive	3.21	0.031^d^	1.11	9.25
*TLR2-SNP 10540*	*rs43706433*	Recessive	2.51	0.020^d^	1.15	5.48
*TLR4-SNP 9788*	*rs8193069*	Additive	0.27^b^	0.026	0.09	0.86
*TLR6-SNP 14578*	*rs43702941*	Additive	2.58^b^	0.012^e^	1.23	5.43
*TLR10-SNP 774*	*rs55617325*	Additive	0.53^b^	0.041^e^	0.29	0.97

a 95% Confidence interval for odds ratio.

b Odds ratio adjusted for the effect of birth year.

c *P*-value not corrected for multiple comparisons.

d *P*-value marginal (0.053) after locus-specific FDR correction [45] (http://sdmproject.com/utilities/?show=FDR).

e *P*-value<0.05 after locus-specific FDR correction [45] (http://sdmproject.com/utilities/?show=FDR). *TLR1*, *TLR6*, and *TLR10* were considered a single locus for multiple test correction.

## Discussion

Our methodological workflows resulted in the robust identification of SNPs with precise estimates of MAF for the bovine *TLR* genes (see methods), as evidenced by the regression of MAFs derived from the analysis of pyrosequencing data and allele-specific genotyping assays (Figure 1). For these genes, our genotyping assays provide a 70 fold increase in marker density relative to the Illumina BovineSNP50 assay, which queries four SNPs either within (*TLR6*, *TLR10*) or proximal to (*TLR7*, *TLR8*) the targeted loci, and a greater than 3 fold increase in marker density relative to the new Illumina BovineHD assay (777K), which possesses an average marker interval density of approximately 1 SNP/3.5 kb. Notably, the new BovineHD assay includes 84 SNPs that are either within or proximal to (≤2 Kb) the 10 *TLR* genes (i.e. *TLR1* [3]; *TLR2* [6]; *TLR3* [8] *TLR4* [6]; *TLR5* [22]; *TLR6* [23]; *TLR7* [3]; *TLR8* [4]; *TLR9* [5]; *TLR10* [4]), including one SNP implicated by our case-control study (*TLR2*-rs43706433; Table 5). Validated polymorphisms, reconstructed haplotypes, and the tagSNPs/Indels identified in this study will directly facilitate the fine mapping of bovine health-related QTL [23]-[27], while also enabling further evaluation of SNPs tentatively associated with differential susceptibility to Johne's disease (MAP infection) [19]-[22], [46] (Table 5). While large numbers of tightly clustered SNPs are sometimes difficult to genotype, we endeavored to validate all detected variants by redesigning primers and manipulating PCR conditions for problematic markers. Accordingly, we successfully validated several SNPs for which assays had previously failed [30], and we also validated the majority of the newly identified putative SNPs (pyrosequencing data) that were not associated with homopolymer repeats. Furthermore, some regions of *TLR1* posed the greatest technical challenge due to sequence similarity with *TLR6*. For this reason, at least some DNA sequencing from medium-range PCR products designed to specifically amplify each locus is needed to exhaustively ascertain all possible variants spanning the *TLR1*-*TLR6* gene cluster.

Across all adjacent variable sites within the bovine *TLR* gene family, we observed higher levels of LD (r^2^) in *B. t. taurus* cattle (0.32) than in the combined sample (0.26) of *Bos t. taurus*, *Bos t. indicus*, and composite breeds (Table 2). This is generally consistent with previous studies of bovine subspecific divergence, haplotype structure, and LD across short to moderate physical distances [3], [47], including our previous study on bovine *TLR* haplotype structure [30]. However, in this study intragenic estimates of r^2^ increased for several loci upon pooling (all cattle), including *TLR4*, *TLR8*, and *TLR10*, which was not predicted given previously reported trends in LD [3], [30], [47]. We previously found that r^2^ values were enhanced after pooling only for *TLR7* and *TLR8* [30]. This result indicates that phase-relationships have been preserved across bovine subspecies and specialized breeds for these loci, perhaps due to selection (Table 4), and is only apparent at high genotyping densities. Moreover, this observation may represent a signature of selection on some individual variable sites, with detectable levels of intragenic selection only becoming apparent (Table 4) with increasing numbers of variable sites subject to selection, and/or uniformly higher selection coefficients. For all genes except *TLR2* (Network 1 only), *TLR3* (Network 1 only), *TLR5*, *TLR8*, and *TLR9*, one or two predominant haplotypes were predicted for the majority of the cattle investigated (Figures 2,3,4, Figure S1; Table S4). Moreover, significantly positive values for Tajima's *D* were detected for genomic regions encoding *TLR3* and *TLR8* (Table 4) despite correction for multiple testing, and for *TLR3*, the addition of best haplotype pairs for sires with phase probabilities<0.90 produced very similar test statistics (*D*) for *B. t. taurus* cattle, indicating that *D* is not falsely inflated by the absence of rare alleles within the sires that could not be stringently phased. Additionally, a regression based test also demonstrated that *TLR3* and *TLR8* possess significantly more diversity than do all other *TLR* loci (*P*≤0.05; Figure 5). Significantly positive values for Tajima's *D* are often interpreted as evidence for a recent population bottleneck, or for some form of balancing selection [48]-[50], with *D* being the most powerful test in its class [51], but may also indicate violations of the mutation-drift equilibrium assumption or random sample requirement. Worthy of discussion is the fact that variation within *TLR3* displayed the second highest average r^2^ values between adjacent variable sites (Table 2), which in conjunction with a large, significantly positive *D* statistic for taurine cattle (Table 4) suggests that this gene is under selection. However, unlike *TLR8*, high r^2^ (≥0.50 for 10/13 SNPs in *TLR8*) did not persist across the majority of all adjacent variable sites in *TLR3*, and therefore, it is relatively unsurprising that our analysis of *TLR3* revealed no evidence for a deficiency of total discrete haplotypes in *B. t. taurus* cattle (i.e., *F_S_* was not significant).

Surprisingly, the region of *TLR3* demonstrating the strongest deviation from neutrality does not include the two nonsynonymous SNPs predicted to impact protein function (Table 3, Table 4), but includes a 5′ putative promoter region (PROSCAN 1.7: http://www-bimas.cit.nih.gov/molbio/proscan/index.html) [23] harboring several transcription factor binding sites (NF-κB, PEA1, AP-1, TFIID; Positions 2852041-2852291 of NW_001494406.2) as well as the first two exons and introns of *TLR3*. No variation was detected within the predicted promoter itself. However, 40 validated SNPs were found to flank the putative promoter (see Table S2 for coordinates), with nearly half of this variation occurring immediately upstream (n = 19 SNPs). Further evaluation of LD between adjacent variable sites for taurine cattle revealed two regions of *TLR3* with persistent, unbroken r^2^>0.50 between all adjacent sites as follows: 1) Variable sites 1-5 upstream of the predicted promoter (Table S2); and 2) Variable sites 10-19, which span the predicted promoter. This unbroken pattern of persistent r^2^ was also detected in our pooled analysis of all cattle, but did not extend across as many adjacent variable sites (Table S2, sites 13-17; region also spans the predicted promoter), and was only found in one upstream region. Therefore, it is possible that selection is primarily operating on noncoding variation within the genomic regions flanking the predicted promoter. Future functional studies will be needed to determine whether the SNPs flanking the predicted *TLR3* promoter actually modulate differences in gene expression.

Notably, only *TLR8* displayed a significant, positive value for Fu's *F_S_*, indicating a lower than expected number of haplotypes, as would be predicted given a recent population bottleneck or strong balancing selection. However, the high r^2^ that persists across nearly all adjacent variable sites strongly implies selection (Table 2). While previous studies have suggested that population bottlenecks may have occurred at the time of domestication and breed formation for modern cattle [3], [47], these are expected to drive frequency distribution tests (*D*, *F_S_*) toward more positive values because of the loss of rare genetic variation at all loci. In particular, the effects of bottlenecks are expected to be uniform and potentially dramatic for proximal, evolutionarily related X-linked loci (*TLR7*, *TLR8*) performing similar functions (6, 11-12), especially given smaller effective population size (chromosomal) and female limited recombination. However, *TLR7* possesses a fundamentally different frequency distribution trend (*D* = -0.19828 all cattle; *D* = -0.17037 *B. t. taurus*) as compared to *TLR8* (*TLR7*≤103 Kb from *TLR8*; Btau5.2), with no evidence for a significant deviation from a strictly neutral model (Table 4). A regression based test also provided no evidence for the effects of a population bottleneck or selection operating on variation within *TLR7* (*P*≥0.05; see Figure 5). Therefore, it seems unlikely that historic bottlenecks are responsible for deviations from neutrality for bovine *TLR8*, and more likely that balancing selection is operating to preserve a limited number of functionally divergent haplotypes. Interestingly, the haplotypes observed for *TLR8* were partitioned into two main functional groups, as classified by our AA modeling (Table 3) and median joining haplotype networks (Figure 3). Specifically, haplotypes that fell into network nodes A, B, and C differed from haplotypes falling into nodes D, E, and F by eight nonsynonymous SNPs encoding AA substitutions (Table S2), with at least two (S477N; K903T) that were predicted to impact protein function (Table 3; Figure 3). Additionally, the four most common haplotypes (nodes A, B, D, and E) differed only by one synonymous SNP (nodes A vs B; encoding S10S) and one putatively benign or tolerated nonsynonymous SNP (nodes D vs E; encoding S492N; see Table S2; Table 3). For these reasons, functional studies are now needed to comprehensively assess the dynamic range of ligand-induced *TLR8* signaling in domestic cattle.

In addition to *in silico* determined signatures of selection, we also provide evidence for associations between several bovine *TLR* SNPs and differential susceptibility to the causative agent of Johne's disease (Table 5). Unlike most previous studies [19]-[22], [46], we detected associations for which *TLR* variation both enhanced and decreased the risk of MAP infection. Furthermore, the SNPs demonstrating associations in this study (Table 5) were within bovine *TLR* genes that are either known or postulated to recognize ligands that would facilitate MAP detection and signaling [7], [11], [12], [19]-[22], [46], [52]. While two recent genome wide association studies (GWAS) employing the Illumina BovineSNP50 assay provided no evidence for *TLR* involvement in differential susceptibility to Johne's disease in cattle [53], [54], the stringency of multiple testing employed during GWAS may have failed to identify *TLR* loci modulating relatively small effects. Moreover, the marker density of the BovineSNP50 assay is insufficient to detect all possible associations with bovine *TLR* variation [30] (Table S2). The SNP density for the new Illumina BovineHD assay also may not be sufficient to detect all disease associations with *TLR* loci, and therefore, additional association and functional studies are needed to clarify the involvement of *TLR2*, *TLR6*, and *TLR10* with respect to differential susceptibility to MAP infection in Holstein cattle.

### Conclusions

Our detailed analysis of the haplotype structure, LD architecture, and tagSNP/Indel prediction for all 10 bovine *TLR* genes will enable studies aimed at assessing the statistical and functional relationships between validated variation, and differential susceptibility to infectious disease [19]–[27], [46] (Table 5). Moreover, because extensive haplotype sharing was confidently predicted for specialized beef and dairy cattle breeds, the deliverables of this study will broadly impact many facets of bovine health research, including the potential for marker-assisted vaccination; using genotypes as indicator variables for enhanced vaccine design or as predictors of animal response.

In view of the emerging global interest in genomic selection in beef and dairy cattle, we provide evidence for balancing selection on at least two of the *TLR* genes (*TLR3* and *TLR8*), with detection of a weaker selective signal consistent with purifying selection in *TLR10* [30] (Table 4). Interestingly, *TLR3* and *TLR8* encode molecular sentries that recognize invading double-stranded (ds) and single-stranded (ss) RNA viruses, respectively, thereafter eliciting host innate immune responses (11, 12). Importantly, selection on *TLR3* and *TLR8* may have direct implications on aspects of differential susceptibility to major viral production diseases such as bluetongue (dsRNA; *Reoviridae*), foot and mouth disease (ssRNA; *Picornaviridae*), bovine viral diarrhea (ssRNA; *Flaviviridae*), calf coronavirus (ssRNA; neonatal diarrhea; *Coronaviridae*), and bovine parainfluenza 3 (ssRNA; *Paramyxoviridae*) (see [55], [56]). Moreover, evolution under repeated exposure to many of these diseases may provide some explanation for the observed patterns of variation detected within *TLR3* and *TLR8*. However, it is also possible that more ancient host-pathogen interactions (i.e., eradicated Rinderpest, ssRNA, *Paramyxoviridae*; etc) may have contributed to the signatures of selection detected in this study. It should also be noted that because frequency distribution tests generally lack power to detect selection [51], departures from neutrality noted in this study are likely to underscore the strength of the selective signals observed (for review see [57]). For these reasons, future studies involving all species of the subfamily Bovinae are needed to help elucidate whether selective signals in *TLR3* and *TLR8* extend beyond modern domestic cattle lineages. Moreover, variation within these genes should be comprehensively evaluated with respect to differences in ligand-induced signaling, disease susceptibility, and the potential for marker-assisted vaccination in domestic cattle.

In addition to selective signals observed for *TLR3* and *TLR8*, several tentative associations were detected between bovine *TLR* SNPs (Table 5) and differential susceptibility to MAP infection which have not previously been reported, with one implicated locus (*TLR10*) also exhibiting evidence of purifying selection (Table 4) [30]. However, because the natural ligand(s) for *TLR10* have yet to be comprehensively elucidated, the precise origin of this selective signal remains unclear. Previous studies [13], [58] indicate that human TLR10 forms functional heterodimers with both TLR2 and TLR1, thereby enabling the resulting protein complexes to recognize a wide variety of microbial ligands [58], including those derived from Mycobacteria [11], [12], [14], [59]. Similarly, TLR2 is also known to form functional heterodimers with TLR6 [14]. Recently, AA substitutions in human *TLR1* and *TLR10* were demonstrated to negatively impact receptor function [58]-[59], with *TLR10* ligand recognition similar to the known range of ligands established for TLR1 [58]. The results of our single marker association tests indirectly support the biological concept of functional unity with respect to bovine *TLR2*, *TLR6*, and *TLR10*, with variation at all three loci categorically linked to a common microbial phenotype (bacterial culture status for MAP) in Holstein cattle.

## Methods

### DNA Samples for SNP Discovery

Bovine DNA samples (n = 96) representing *B. t. taurus*, *B. t. indicus*, and their hybrids were isolated from spermatozoa as previously described [23], [25], [30]. Bovine subspecies designation, breed names, and sample sizes (in parentheses) were: *B. t. taurus* - Angus (5), Belgian Blue (2), Blonde d'Aquitaine (1), Braunvieh (4), Brown Swiss (2), Charolais (6), Chianina-Chiangus (4), Corriente (1), Gelbvieh (4), Hereford (3), Holstein (6), Limousin (4), Maine-Anjou (3), Red Angus (4), Red Poll (1), Salers (2), Senepol (2), Shorthorn (4), Simmental (5), Texas Longhorn (2); *B. t. indicus* - Brahman (8), Nelore (2); Hybrids, termed Composites - Beefmaster (4), Braford (2), Brahmousin (2), Brangus (3), Piedmontese (1), Red Brangus (2), Romagnola (2), Santa Gertrudis (2), Simbrah (3). Bovine subspecies were assigned based on phenotype and breed origin (http://www.ansi.okstate.edu/breeds/cattle/).

### Bovine *TLR* Sequencing and SNP Detection

Procedures involving primer design**,** PCR amplification with gene-specific primers, and standard dye-terminator cycle sequencing (Sanger) of all 10 bovine *TLR*s have previously been described [23]-[25], [60]. For this study, we synthesized gene-specific amplification primers with a unique 10 bp 5′ barcode (Roche MIDs) for each of the 10 bovine *TLR* genes (Table S5). Thereafter, we standardized all 96 discovery panel DNAs to 50 ng/µl and created three DNA pools, with each pool consisting of 32 elite sire DNAs mixed at equal concentrations. Notably, larger-scale DNA pooling in a human amplicon study supports the accuracy and reliability of this approach when coupled with Roche 454 pyrosequencing [61]. Three bovine DNA pools were used to amplify all *TLR* targets via barcoded primers (Table S5), with PCR conditions and thermal parameters as previously described [23]-[25], [60]. Targets that were intolerant to the addition of 5′ oligonucleotide barcodes for PCR amplification were amplified using standard primers in conjunction with downstream dye-terminator cycle sequencing methods previously described [23]-[25], [60], with one exception: A second set of DNA pools (n = 12) was created, with each pool containing equal concentrations of DNA from eight elite sires derived from the sequencing discovery panel. Importantly, both sets of DNA pools (Sanger and Roche 454) were seeded with one or more reference DNAs that had previously been sequenced and/or SNP genotyped across all 10 bovine *TLR* genes [23]-[25], [60], which collectively included≥12 reference DNAs possessing 216 validated diallelic variants (212 SNPs + 4 indels) [30]. All amplicons were purified using the Qiaquick PCR purification kit (Qiagen, Valencia, CA) as previously described [24], [25], and the concentrations were estimated by Nanodrop. For preparation of a Roche 454 Titanium fragment library, we standardized all barcoded amplicons to 10 ng/µl and devised a normalization procedure that accounted for differences in amplicon size (Table S1). Because the *TLR* amplicons differed in size, an adjustment was necessary to ensure balanced 454 pyrosequencing results. Specifically, using amplicon size, we computed the mean (bp) and standard deviation (SD; bp) across all PCR targets. Thereafter, any amplicon deviating from the mean by≥0.5 SDs in either direction was subject to proportional adjustment within the fragment library (Table S1). The direction of adjustment (plus or minus) was determined by the direction of the deviation (i.e., smaller = proportionally less template; larger = proportionally more template; Table S1). Because the emulsion PCR process involved in the preparation of Roche 454 Titanium fragment libraries favors smaller fragments, amplicons smaller than the mean by≥0.5 SDs must be proportionally reduced in the final library, whereas the opposite is true for larger amplicons. Following normalization, the bovine *TLR* sequencing library was constructed via random ligation of sequencing adaptors provided with the GS FLX Titanium library kit (Roche Applied Science, Indianapolis, IN). All library preparation, emulsion PCR, quantitation, and sequencing steps followed the manufacturer's protocol (Roche Applied Science).

SNP detection analyses for the resulting pyrosequencing data employed the Neighborhood Quality Standard algorithm [62], [63] implemented within CLC Genomics Workbench (v3.7.1), as previously described [29]. Putative SNPs were filtered using a method devised from *a priori* knowledge of biallelic controls (212 SNPs + 4 indels) [30] that were purposely seeded into the amplicon library. Briefly, we considered the possibility that some SNPs may only be found as one allele in a single elite sire (1/192 total alleles; see reference 30 for examples). Therefore, we filtered all putative SNPs predicted from our analysis of the pyrosequencing data using the following formula: 1/192×(Total SNP Coverage) = Theoretical minimum number of reads, which represents the smallest number of reads required to shuttle putative SNPs into a validation workflow involving custom, allele-specific genotyping assays. This method proved valuable for the discovery and validation of many low frequency SNPs, including those that occurred as one allele for a single discovery panel sire (i.e., *TLR5* putative nonsense SNP = 1/192 alleles in the discovery panel). For SNP discovery using standard dye-terminator sequencing reads, we used an alignment-based method of variant detection within the program Sequencher 4.6 [23], [25]. Briefly, high quality electropherograms were manually inspected for any evidence of a double peak. Individual nucleotide sites displaying any evidence of heterozygosity within≥1 sequencing read were shuttled to our SNP validation workflow.

### SNP Validation and Genotyping

All 96 DNAs from the pyrosequencing discovery panel were also used for allele-specific genotyping. Additionally for bovine *TLR*s recognizing bacterial ligands, we also utilized the following industry-relevant DNA panels: Beef (48 Purebred Angus, 1 Herd); Dairy (405 Holstein dairy cows, 3 Herds). SNPs and indels were genotyped using the KASPar allele-specific fluorescent genotyping system (Kbiosciences, Hertfordshire UK), as previously described [29], [30]. Thermal cycling parameters and reaction concentrations followed manufacturer's recommendations, with some modifications to MgCl_2_ concentrations. Primer sequences and MgCl_2_ concentrations are available on request. Genotype clustering and calling was performed using KlusterCaller software (Kbiosciences). Genotype quality was assessed by manually inspecting the clustering data for every individual marker, and by comparing KASPar-derived genotypes to those derived from previously reported sequence data [23], [25], [30]. Poor clustering or inconsistent genotypes precipitated the following workflow: 1) Further optimization and/or redesigning the SNP assay followed by; 2) Genotyping the inconsistent samples again. Notably, to minimize the frequency of missing genotypes from a very low proportion of failed assays, most SNPs were genotyped multiple times for every DNA sample. Genotype data are available in Table S6.

### Haplotype Inference, LD Estimates and Variant Tagging

Unphased diploid genotypes were compiled and cross-checked for parsing errors using two custom software packages [30]. Haplotype reconstruction and missing data imputation (<0.58%) was performed with PHASE 2.1 [31], [64], [65] using all validated intragenic polymorphisms, all cattle for a given locus, and the –X10 option. Haplotype estimation using PHASE 2.1 is not sensitive to departures from HWE 31,64,65. Predicted haplotype phases with best pair probabilities≥0.90 were retained for further analysis. Bovine X-linked haplotypes (*TLR7*, *TLR8*) were directly ascertained by genotype homozygosity in our sire panel used for SNP discovery. Estimates of recombination across each gene were also assessed in PHASE 2.1 using the general model for varying recombination rate [31], [34], [35]. Deviation from the average background recombination rate () [34], [35] by a factor≥2.5 between adjacent sites was considered evidence for historical recombination.

Intragenic LD was visualized within Haploview [32] using unphased diploid autosomal genotypes and phase-known X-linked data (*TLR7*, *TLR8*) for *B. t. taurus* samples, and all cattle combined. LD patterns and blocks were estimated via majority rule from: 95% confidence intervals constructed for D'[32], [33]; application of the four gamete rule [32] (4^th^ gamete>0.02); and estimates of recombination between adjacent sites [34], [35]. To further evaluate patterns of LD decay, pairwise r^2^ values were estimated with Haploview for all validated markers within each gene for *B. t. taurus* and all cattle combined. A minimal set of tagSNPs/Indels predicted to capture 100% of the variation (r^2^>0.80) segregating in *B. t. taurus* and all cattle combined was deduced using the Tagger algorithm implemented in Haploview.

### Median Joining Haplotype Networks

Because median joining (MJ) networks require the absence of recombination [66], genes displaying evidence of historical recombination (*TLR2*, *TLR3*, *TLR6*) were each partitioned into two regions of elevated LD. Haplotypes were reconstructed [31] for each intragenic region and best pairs were used for MJ network analyses [28]. This approach improved the proportion of cattle with best pairs phase probabilities≥0.90 and eliminated regions displaying overt evidence of recombination. MJ networks were constructed using Network 4.5.1.0 (Fluxus Technology Ltd, Suffolk, England), and the default character weights of 10 for SNPs and 20 for indels. Results were visualized, annotated, and adjusted within Network Publisher (Fluxus Technology Ltd, Suffolk, England). Branch angles were adjusted to ensure proper network magnification and clarity without changing branch lengths.

### AA Substitution Phenotypes and *TLR10* Evolutionary Analyses

Bovine AA substitution phenotypes were predicted using PolyPhen [38] and SIFT [39] (http://genetics.bwh.harvard.edu/pph/; http://genetics.bwh.harvard.edu/pph/pph_help.html; http://sift.jcvi.org/; http://sift.jcvi.org/www/SIFT_help.html) with the default settings. Results other than “benign” or “tolerated” were categorized as substitutions predicted to impact protein function [30], [38], [39]. To assess the potential for functional and/or selective constraint across the entire bovine *TLR* gene family, a goodness of fit test (χ^2^) was performed assuming equal probabilities for benign or tolerated AA phenotypes versus those predicted to impact protein function. Frequency distribution tests, including Tajima's *D* [43] and Fu's *F*_S_ [44], were performed in DnaSP v4.90.1 [67] using all validated SNPs. Significance levels for frequency distribution tests were defined by confidence intervals estimated for each test statistic via coalescent simulation (10,000 replicates) [67]. Simulations were performed given the observed number of segregating sites, both with and without recombination [67], [68].

At each polymorphism we estimated the effective number of alleles as E_i_ = 1/(1 - 2p_i_(1-p_i_)) = 1/(p_i_^2^ + (1 - p_i_)^2^) = 1/(expected HWE frequency of homozygotes) where p_i_ is allele frequency at the i^th^ locus. Thus a measure of polymorphism diversity is log_2_(E_i_) which also represents the information content of each SNP [30]. For monomorphic SNPs log_2_(E_i_) = 0 and for SNPs with p_i_ = 0.5, log_2_(E_i_) = 1. Thus by summing across the N_j_ polymorphisms within the j^th^ gene we obtain the diversity index I_j_ =  . We used regression analysis to examine the relationship between I_j_ and N_j_ for these genes and to test for outliers using 95% confidence estimates for the fitted regression.

### Association Tests with MAP infection status

A case-control study was performed to estimate the association between specific *TLR* genotypes and MAP infection in Holstein cattle. The study population was derived from an established repository [69] that included whole blood samples preserved from adult Holstein cattle in three herds that were characterized on the basis of: 1) MAP bacterial culture of feces; 2) MAP bacterial culture of tissues for harvested cattle; 3) ELISA values for MAP-specific antibody. Cattle from which MAP was cultured in the feces and/or the tissues collected at harvest were selected as cases (n = 68). Herd-matched controls (n = 270) were selected from those cattle in the repository with negative ELISA and bacterial culture data. Cattle with multiple negative tests were preferentially selected to reduce the probability of misclassification relative to infection status due to the low sensitivity of available diagnostic methods for MAP. DNA was extracted from available blood specimens using a commercial kit (MoBio DNA non-spin, Carlsbad, CA) and assessed for quality as well as concentration by standard spectrophotometric methods. Genotypes for validated SNPs and indels in the 5′ upstream regions, introns, and those associated with nonsynonymous or putative nonsense mutations in bovine *TLR* genes recognizing bacterial ligands (*TLR1*, *TLR2*, *TLR4*, *TLR5*, *TLR6*, *TLR9*, *TLR10*) (see refs [11], [14]) were evaluated for further analysis. Loci fixed for the major allele in our dairy population were excluded, leaving 35 nonsynonymous and 1 putative nonsense substitution, and 37 other SNP loci within the 5′ upstream regions or intragenic introns. For these 73 variable sites, we excluded SNPs and indels with MAFs<0.01 in our infected cases, leaving 32 SNPs and 3 indels for association tests (see Table S1).

Conditional logistic regression models were constructed for each of the 35 variable loci to estimate the relative odds of being infected with MAP based on the defined diagnostic criteria adjusted for the effects of herd using the PHREG procedure of SAS (SAS v. 9.2, SAS, Cary, NC). Effects of genotype were estimated using 3 different covariate specifications. First, an additive mode of inheritance was examined whereby the odds of infection associated with each additional copy of the minor allele was modeled as a single continuous covariate. Second, a recessive mode of inheritance was modeled, where the odds of infection in cattle homozygous for the minor allele were estimated relative to cattle heterozygous and homozyzgous for the major allele. Finally, each genotype was modeled as an indicator variable and effect estimates were generated for cattle homozygous for the minor allele, and for heterozygous cattle, both relative to cattle homozygous for the major allele. This allowed evaluation of assumptions in the additive model with respect to the effect of the additional copies of the minor allele being linear in the log odds, and potential intermediate effects of the minor allele not captured in the other models. Potential confounding by age was examined by including birth year as a fixed covariate (where available), and was defined as a change in the relative odds of greater than 20% after addition of the birth year term. For models where evidence of confounding by age was detected, birth year was retained in the model to adjust genotype estimates for this effect. With the exception of *TLR1*, *TLR6*, and *TLR10*, all single marker *P*-values were corrected for multiple testing by applying the FDR correction (http://sdmproject.com/utilities/?show=FDR) [45] to the raw *P*-values derived from each investigated gene (locus-specific correction). Given the close physical proximity of *TLR1*, *TLR6*, and *TLR10* on BTA6, these genes were considered a single locus for correction of multiple tests. However, it should be noted that none of the variable markers within *TLR1* met our inclusion criteria (MAFs>0.01), and therefore, locus-specific correction was only applied to raw *P*-values from *TLR6* and *TLR10*.

Haplotype association tests were performed in PHASE 2.1 [31]. Briefly, for dairy cattle with disease classifications based on bacterial culture status of MAP, we tested the hypothesis that haplotypes differ among cases and controls for all genes evaluated in the single marker association analysis (68 cases, 270 controls, n = 338 total). For maximum LD-based resolution of haplotypes, we used all variable markers within seven bovine *TLR* genes that recognize bacterial ligands. Significance was estimated via 1,000 permutations.

## Supporting Information

Figure S1 **Median joining (MJ) haplotype networks constructed for bovine** ***TLR1, TLR2, TLR4, TLR5, TLR6, TLR7, and TLR9*** **using haplotypes predicted for all cattle.** For all loci except TLR7, all cattle is defined as follows: n = 96 AI sires, 31 breeds; 48 Purebred Angus; 405 Holstein cattle. For TLR7, only the sequencing discovery panel was genotyped and is represented (n = 96 AI sires, 31 breeds). Because MJ networks require the absence of recombination [66], each network represents intragenic regions of elevated LD. Haplotypes predicted for *B. t. taurus*, *B. t. indicus* and hybrids (termed “composites”) are color coded. Numbers indicate SNP and indel positions in numerical order (see Table S2 for SNP information). Node sizes are proportional to haplotype frequency, and all branch lengths are drawn to scale. Alphabetized letters at nodes represent the breed distribution of each haplotype (Table S4). Median vectors are indicated as “mv”.
(PPTX)

Table S1 ***TLR*** **Amplicon Normalization (XLSX).**
(XLSX)

Table S2 **Validated SNPs and Indels (XLSX).**
(XLSX)

Table S3 **TagSNPs and Indels (XLSX).**
(XLSX)

Table S4 **Network Node Breed Key (XLSX).**
(XLSX)

Table S5 **Barcoded Primers (XLSX).**
(XLSX)

Table S6 ***TLR*** **Genotype Data (XLSX).**
(XLSX)

## References

[1] VanRaden PM, Van Tassell CP, Wiggans GR, Sonstegard TS, Schnabel RD (2009). Invited review: reliability of genomic predictions for North American Holstein bulls.. J Dairy Sci.

[2] Bovine Genome Sequencing, Analysis Consortium, Elsik CG, Tellam RL, Worley KC (2009). The genome sequence of taurine cattle: A window to ruminant biology and evolution.. Science.

[3] Bovine HAPMAP Consortium (2009). Genome-Wide Survey of SNP Variation Uncovers the Genetic Structure of Cattle Breeds.. Science.

[4] Rosenthal KL (2006). Tweaking innate immunity: The promise of innate immunologicals as anti-infectives.. Can J Infect Dis Med Microbiol.

[5] Vasselon T, Detmers PA (2002). Toll Receptors: a Central Element in Innate Immune Responses.. Infect Immun.

[6] Kaisho T, Akira S (2006). Toll-like receptor function and signaling.. J Allergy Clin Immunol.

[7] Plain KM, Purdie AC, Begg DJ, de Silva K, Whittington RJ (2010). Toll-like receptor (TLR) 6 and TLR1 differentiation in gene expression studies of Johne's disease.. Vet Immunol Immunopathol.

[8] Jann OC, King A, Corrales NL, Anderson SI, Jensen K (2009). Comparative genomics of Toll-like receptor signaling in five species.. BMC Genomics.

[9] Kataria RS, Tait RG, Kumar D, Ortega MA, Rodiguez J (2011). Association of toll-like receptor four single nucleotide polymorphisms with incidence of infectious bovine keratoconjunctivitis (IBK) in cattle.. Immunogenetics.

[10] Glass EJ, Baxter R, Leach RJ, Jann OC (2011). Genes controlling vaccine responses and disease resistance to respiratory viral pathogens in cattle..

[11] West AP, Koblansky AA, Ghosh S (2006). Recognition and Signaling by Toll-Like Receptors.. Annu Rev Cell Dev Biol.

[12] Akira S, Takeda K (2004). Toll-like receptor signaling.. Nat Rev Immunol.

[13] Hasan U, Chaffois C, Gaillard C, Saulnier V, Merck E (2005). Human TLR10 is a functional receptor, expressed by B cells and plasmacytoid dendritic cells, which activates gene transcription through MyD88.. J Immunol.

[14] Ozinsky A, Underhill DM, Fontenot JD, Hajjar AM, Smith KD (2000). The repertoire for pattern recognition of pathogens by the innate immune system is defined by cooperation between Toll-like receptors.. Proc Natl Acad Sci U S A.

[15] Mukhopadhyay S, Herre J, Brown GD, Gordon S (2004). The potential for Toll-like receptors to collaborate with other innate immune receptors.. Immunology.

[16] Govindarai RG, Manavalan B, Lee G, Choi S (2010). Molecular modeling-based evaluation of hTLR10 and identification of potential ligands in Toll-like receptor signaling. PLoS ONE 5(9):e12713.. http://www.ncbi.nlm.nih.gov/pmc/articles/PMC2943521/?tool=pubmed.

[17] Merx S, Zimmer W, Neumaier M, Ahmad-Nejad PA (2006). Characterization and functional investigation of single nucleotide polymorphisms (SNPs) in the human TLR5 gene.. Hum Mutat.

[18] Texereau J, Chiche JD, Taylor W, Choukroun G, Comba B (2005). The importance of Toll-like receptor 2 polymorphisms in severe infections.. Clin Infect Dis.

[19] Mucha R, Bhide MR, Chakurkar EB, Novak M, Mikula I (2009). Toll-like receptors TLR1, TLR2, and TLR4 gene mutations and natural resistance to *Mycobacterium avium* subsp. *paratuberculosis* infection in cattle.. Vet Immunol Immunopathol.

[20] Bhide MR, Mucha R, Mukula I, Kisova L, Skrabana R (2009). Novel mutations in TLR genes cause hyporesponsiveness to *Mycobacterium avium* subsp. *paratuberculosis* infection.. BMC Genet.

[21] Pinedo PJ, Buergelt CD, Donovan GA, Melendez P, Morel L (2009). Candidate gene polymorphisms (BoIFNG, TLR4, SLC11A1) as risk factors for paratuberculosis infection in cattle.. Prev Vet Med.

[22] Pinedo PJ, Wang C, Li Y, Rae DO, Wu R (2009). Risk haplotype analysis for bovine paratuberculosis.. Mamm Genome.

[23] Cargill EJ, Womack JE (2007). Detection of polymorphisms in bovine toll-like receptors 3, 7, 8, and 9.. Genomics.

[24] Seabury CM, Cargill EJ, Womack JE (2007). Sequence variability and protein domain architectures for bovine Toll-like receptors 1, 5, and 10.. Genomics.

[25] Seabury CM, Womack JE (2008). Analysis of sequence variability and protein domain architectures for bovine Peptidoglycan Receptor Protein 1 (PGLYRP1) and Toll-Like Receptors 2 and 6.. Genomics.

[26] Kuhn CH, Bennetwitz J, Reinsch N, Xu N, Thomsen H (2003). Quantitative trait loci mapping of functional traits in the German Holstein cattle population.. J Dairy Sci.

[27] Heyen DW, Weller JI, Ron M, Ban M, Beever JE (1999). A genome scan for QTL influencing milk production and health traits in dairy cattle.. Physiol Genomics.

[28] Bandelt HJ, Forster P, Rohl A (1999). Median joining networks for inferring intraspecific phylogenies.. Mol Biol Evol.

[29] Seabury CM, Bhattarai EK, Taylor JF, Viswanathan GG, Cooper SM (2011). Genome-wide polymorphism and comparative analyses in the white-tailed deer (*Odocoileus virginianus*): a model for conservation genomics.. PLoS ONE.

[30] Seabury CM, Seabury PM, Decker JE, Schnabel RD, Taylor JF (2010). Diversity and evolution of 11 innate immune genes in *Bos taurus taurus* and *Bos taurus indicus* cattle.. Proc Natl Acad Sci U S A.

[31] Stephens M, Smith NJ, Donnelly P (2001). A new statistical method for haplotype reconstruction from population data.. Am J Hum Genet.

[32] Barrett JC, Fry B, Maller J, Daly MJ (2005). Haploview: analysis and visualization of LD and haplotype maps.. Bioinformatics.

[33] Gabriel SB, Schaffner SF, Nguyen H, Moore JM, Roy J (2002). The structure of haplotype blocks in the human genome.. Science.

[34] Li N, Stephens M (2003). Modeling linkage disequilibrium and identifying recombination hotspots using single-nucleotide polymorphism data.. Genetics.

[35] Crawford D, Bhangale T, Li N, Hellenthal G, Rieder MJ (2004). Evidence for substantial fine-scale variation in recombination rates across the human genome.. Nat Genet.

[36] Bradley DG, MacHugh DE, Cunningham P, Loftus RT (1996). Mitochondrial diversity and the origins of African and European cattle.. Proc Natl Acad Sci U S A.

[37] Van Tassell CP, Smith TP, Matukumalli LK, Taylor JF, Schnabel RD (2008). SNP discovery and allele frequency estimation by deep sequencing of reduced representation libraries.. Nat Methods.

[38] Ramensky V, Bork P, Sunyaev S (2002). Human non-synonymous SNPs: server and survey.. Nucleic Acids Res.

[39] Kumar P, Henikoff S, Ng P (2009). Predicting the effects of coding non-synonymous variants on protein function using the SIFT algorithm.. Nat Protoc.

[40] Ng PC, Henikoff S (2006). Predicting the effects of amino acid substitutions on protein function.. Annu Rev Genom Human Genet.

[41] Hughes AL, Packer B, Welch R, Bergen AW, Chanock SJ (2003). Widespread purifying selection at polymorphic sites in human protein-coding loci.. Proc Natl Acad Sci U S A.

[42] Subramanian S, Kumar S (2006). Higher intensity of purifying selection on>90% of the human genes revealed by the intrinsic replacement mutation rates.. Mol Biol Evol.

[43] Tajima F (1989). Statistical method for testing the neutral mutation hypothesis by DNA polymorphism.. Genetics.

[44] Fu Y-X (1997). Statistical tests of neutrality of mutations against population growth, hitchhiking, and background selection.. Genetics.

[45] Benjamini Y, Hochberg Y (1995). Controlling the false discovery rate: a practical and powerful approach to multiple testing.. J R Stat Soc Series B.

[46] Ruiz-Larranaga O, Manzano C, Iriondo M, Garrido JM, Molina E (2011). Genetic variation of toll-like receptor genes and infection by *Mycobacterium avium* ssp. *paratuberculosis* in Holstein-Freisian cattle.. J Dairy Sci.

[47] Villa-Angulo R, Matukumalli LK, Gill CA, Choi J, Van Tassell CP (2009). High-resolution haplotype block structure in the cattle genome. BMC Genet..

[48] Hiwatashi T, Okabe Y, Tsutsui T, Hirmatsu C, Melin AD (2010). An explicit signature of balancing selection for color-vision variation in new world monkeys.. Mol Biol Evol.

[49] Tennessen JA, Blouin MS (2008). Balancing selection at a frog antimicrobial peptide locus: fluctuating immune effector alleles?. Mol Biol Evol.

[50] Osier FH, Weedall GD, Verra F, Murungi L, Tetteh KK (2010). Allelic diversity and naturally acquired allele-specific antibody responses to *Plasmodium falciparum* apical membrane antigen 1 in Kenya.. Infect Immun.

[51] Simonsen KL, Churchill GA, Aquadro CF (1995). Properties of statistical tests of neutrality for DNA polymorphism data.. Genetics.

[52] Shey MS, Randhawa AK, Bowmaker M, Smith E, Scriba TJ (2010). Single nucleotide polymorphisms in toll-like receptor 6 are associated with altered lipopeptide-and mycobacteria-induced interleukin-6 secretion.. Genes Immun.

[53] Zanella R, Settles ML, McKay SD, Schnabel R, Taylor J (2010). Identification of loci associated with tolerance to Johne's disease in Holstein cattle.. Anim Genet.

[54] Neibergs HL, Settles ML, Whitlock RH, Taylor JF (2010). GSEA-SNP identifies genes associated with Johne's disease in cattle.. Mamm Genome.

[55] Fauquet C, Mayo MA, Maniloff J, Desselberger U, Ball LA (2005). Virus Taxonomy. London: Elsevier Academic Press.. Pages.

[56] Cahn CM, Line S (2005). The Merck Veterinary Manual. Whitehouse Station: Merck Sharp & Dohme Corp.. Pages.

[57] Bamshad MJ, Mummidi S, Gonzalez E, Ahuja SS, Dunn DM (2002). A strong signature of balancing selection in the 5′ cis-regulatory region of CCR5.. Proc Natl Acad Sci U S A.

[58] Guan T, Ranoa DR, Jiang S, Mutha SK, Li X (2010). Human TLRs 10 and 1 share common mechanisms of innate immune sensing but not signaling.. J Immunol.

[59] Uciechowski P, Imhoff H, Lange C, Meyer CG, Browne EN (2011). Susceptibility to tuberculosis is associated with TLR1 polymorphisms resulting in a lack of TLR1 cell surface expression.. J Leukoc Biol.

[60] White SN, Taylor KH, Abbey CA, Gill CA, Womack JE (2003). Haplotype variation in bovine Toll-like receptor 4 and computational prediction of a positively selected ligand-binding domain.. Proc Natl Acad Sci USA.

[61] Ingman M, Gyllensten U (2009). SNP frequency estimation using massively parallel sequencing of pooled DNA.. Eur J Hum Genet.

[62] Altshuler D, Pollara VJ, Cowles CR, Etten Van WJ, Baldwin J (2000). An SNP map of the human genome generated by reduced representation shotgun sequencing.. Nature.

[63] Brockman W, Alvarez P, Young S, Garber M, Giannoukos G (2008). Quality scores and SNP detection in sequencing-by-synthesis systems.. Genome Res.

[64] Stephens M, Donnelly P (2003). A comparison of Bayesian methods for haplotype reconstruction from population genotype data.. Am J Hum Genet.

[65] Marchini J, Cutler D, Patterson N, Stephens M, Eskin E (2006). A comparison of phasing algorithms for trios and unrelated individuals.. Am J Human Genet.

[66] Posada D, Crandall KA (2001). Intraspecific gene genealogies: trees grafting into networks.. Trends Ecol Evol.

[67] Rozas J (2009). DNA sequence polymorphism analysis using DnaSP.. Methods Mol Biol.

[68] Hudson RR (1987). Estimating the recombination parameter of a finite population model without selection.. Genet Res.

[69] Pradhan AK, Mitchell RM, Kramer AJ, Zurakowski MJ, Fyock TL (2011). Molecular epidemiology of *Mycobacterium avium* subsp. *paratuberculosis* in a longitudinal study of three dairy herds. J Clin Microbiol..

