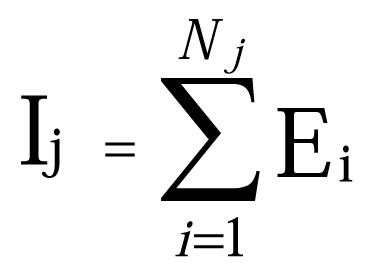# Correction: Evolution of the Bovine *TLR* Gene Family and Member Associations with *Mycobacterium avium* Subspecies *paratuberculosis* Infection

**DOI:** 10.1371/annotation/63a06de9-db62-4651-87f3-a5fd431dcd80

**Published:** 2012-01-12

**Authors:** Colleen A. Fisher, Eric K. Bhattarai, Jason B. Osterstock, Scot E. Dowd, Paul M. Seabury, Meenu Vikram, Robert H. Whitlock, Ynte H. Schukken, Robert D. Schnabel, Jeremy F. Taylor, James E. Womack, Christopher M. Seabury

There is an error in the equation in the "AA Substitution Phenotypes and TLR10 Evolutionary Analyses" section of the Methods. The correct equation can be viewed here: