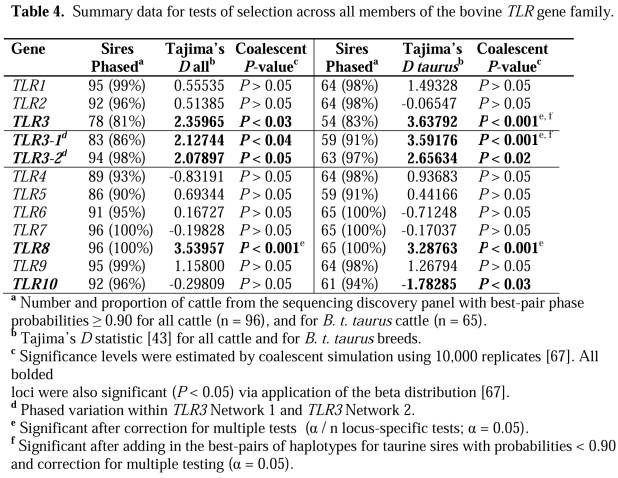# Correction: Evolution of the Bovine *TLR* Gene Family and Member Associations with *Mycobacterium avium* Subspecies *paratuberculosis* Infection

**DOI:** 10.1371/annotation/429afd9a-2892-47ac-a403-008496b2e8db

**Published:** 2012-01-12

**Authors:** Colleen A. Fisher, Eric K. Bhattarai, Jason B. Osterstock, Scot E. Dowd, Paul M. Seabury, Meenu Vikram, Robert H. Whitlock, Ynte H. Schukken, Robert D. Schnabel, Jeremy F. Taylor, James E. Womack, Christopher M. Seabury

There is an error in the formatting of Table 4. Bold formatting is not present. The correct Table 4 can be viewed here: 

**Figure:**