# Correction: Evolution of the Bovine *TLR* Gene Family and Member Associations with *Mycobacterium avium* Subspecies *paratuberculosis* Infection

**DOI:** 10.1371/annotation/a84f0490-55cd-4b42-8b2e-72661fabfc22

**Published:** 2012-02-03

**Authors:** Colleen A. Fisher, Eric K. Bhattarai, Jason B. Osterstock, Scot E. Dowd, Paul M. Seabury, Meenu Vikram, Robert H. Whitlock, Ynte H. Schukken, Robert D. Schnabel, Jeremy F. Taylor, James E. Womack, Christopher M. Seabury

There was a formatting error in the last sentence of the first paragraph of the Haplotype Inference, LD Estimates and Variant Tagging section of Methods. The symbol that should appear in the parentheses can be viewed here: 


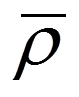


