# Supplementary material for: Evolution of the Bovine TLR Gene Family and Member Associations with Mycobacterium avium Subspecies paratuberculosis Infection
Source: PLoS One. 2011 Nov 30;6(11):e27744. doi: 10.1371/journal.pone.0027744 (PMC3227585; doi:10.1371/journal.pone.0027744)
Supplement: Figure S1 — Median joining (MJ) haplotype networks constructed for bovine TLR1, TLR2, TLR4, TLR5, TLR6, TLR7, and TLR9 using haplotypes predicted for all cattle. For all loci except TLR7, all cattle is defined as follows: n = 96 AI sires, 31 breeds; 48 Purebred Angus; 405 Holstein cattle. For TLR7, only the sequencing discovery panel was genotyped and is represented (n = 96 AI sires, 31 breeds). Because MJ networks require the absence of recombination [66], each network represents intragenic regions of elevated LD. Haplotypes predicted for B. t. taurus, B. t. indicus and hybrids (termed “composites”) are color coded. Numbers indicate SNP and indel positions in numerical order (see Table S2 for SNP information). Node sizes are proportional to haplotype frequency, and all branch lengths are drawn to scale. Alphabetized letters at nodes represent the breed distribution of each haplotype (Table S4). Median vectors are indicated as “mv”. (PPTX) [file pone.0027744.s001.pptx]

## Slide 1
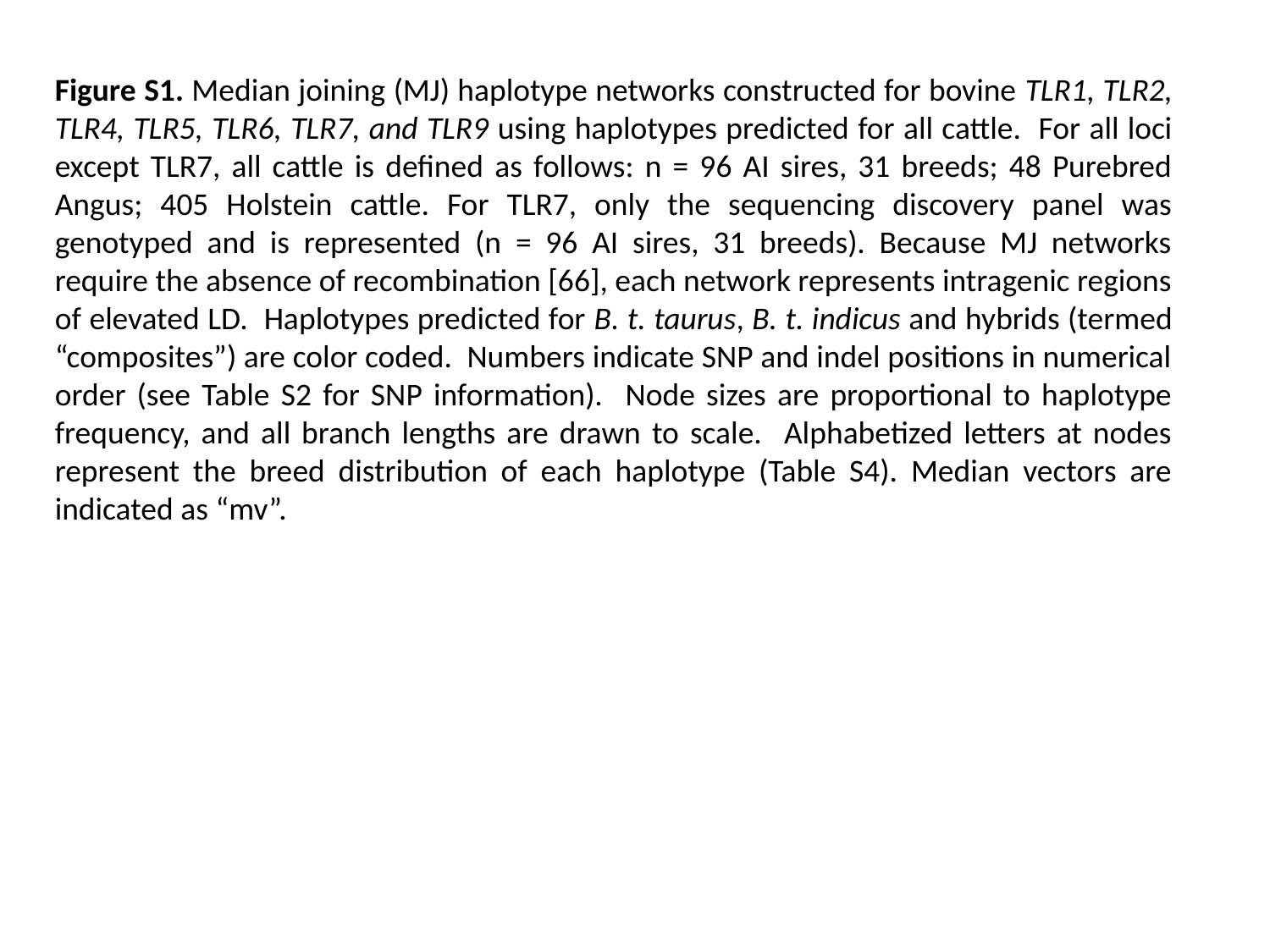

Figure S1. Median joining (MJ) haplotype networks constructed for bovine TLR1, TLR2, TLR4, TLR5, TLR6, TLR7, and TLR9 using haplotypes predicted for all cattle. For all loci except TLR7, all cattle is defined as follows: n = 96 AI sires, 31 breeds; 48 Purebred Angus; 405 Holstein cattle. For TLR7, only the sequencing discovery panel was genotyped and is represented (n = 96 AI sires, 31 breeds). Because MJ networks require the absence of recombination [66], each network represents intragenic regions of elevated LD. Haplotypes predicted for B. t. taurus, B. t. indicus and hybrids (termed “composites”) are color coded. Numbers indicate SNP and indel positions in numerical order (see Table S2 for SNP information). Node sizes are proportional to haplotype frequency, and all branch lengths are drawn to scale. Alphabetized letters at nodes represent the breed distribution of each haplotype (Table S4). Median vectors are indicated as “mv”.

## Slide 2
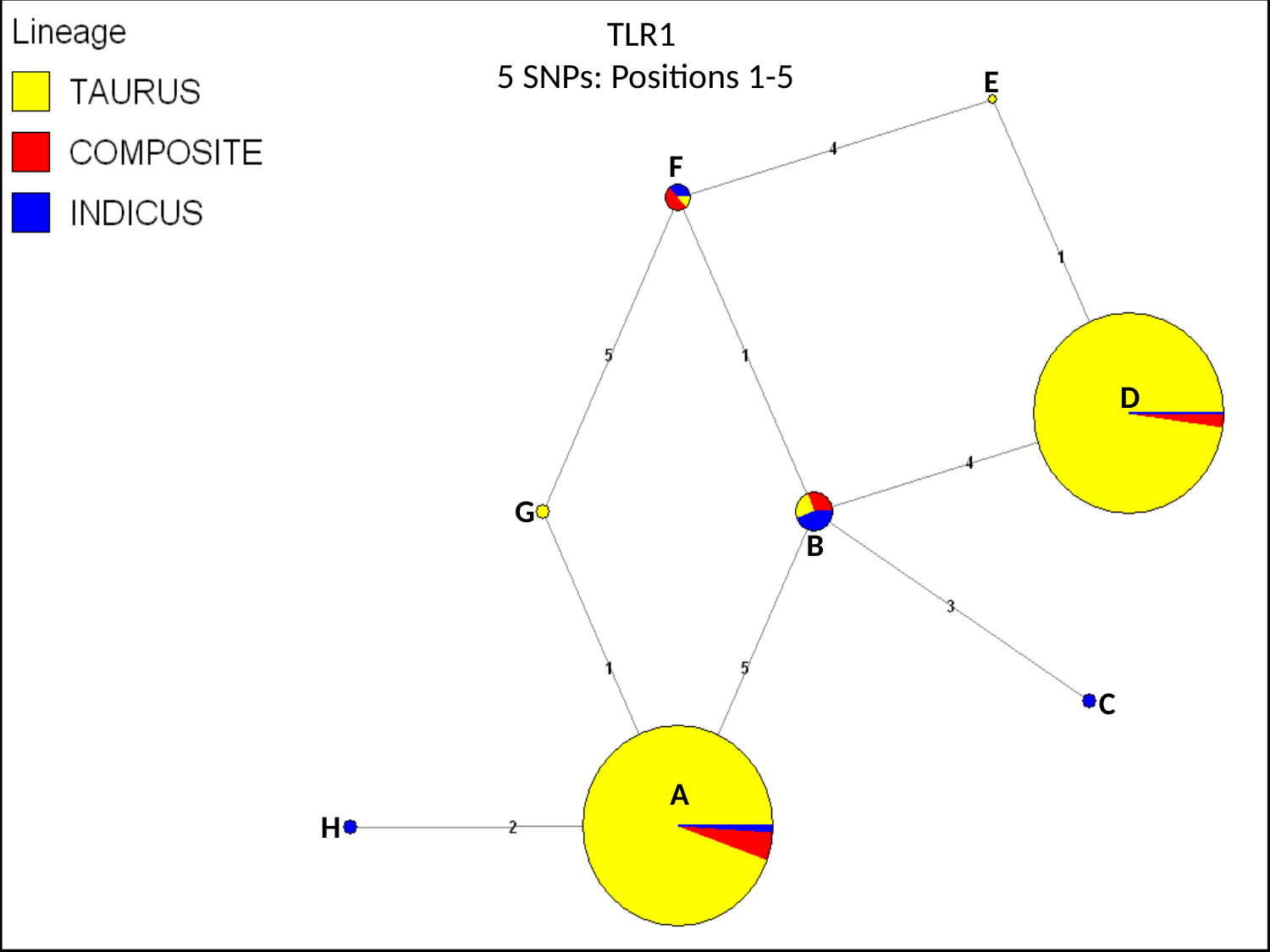

TLR1
5 SNPs: Positions 1-5
E
F
D
G
B
C
A
H

## Slide 3
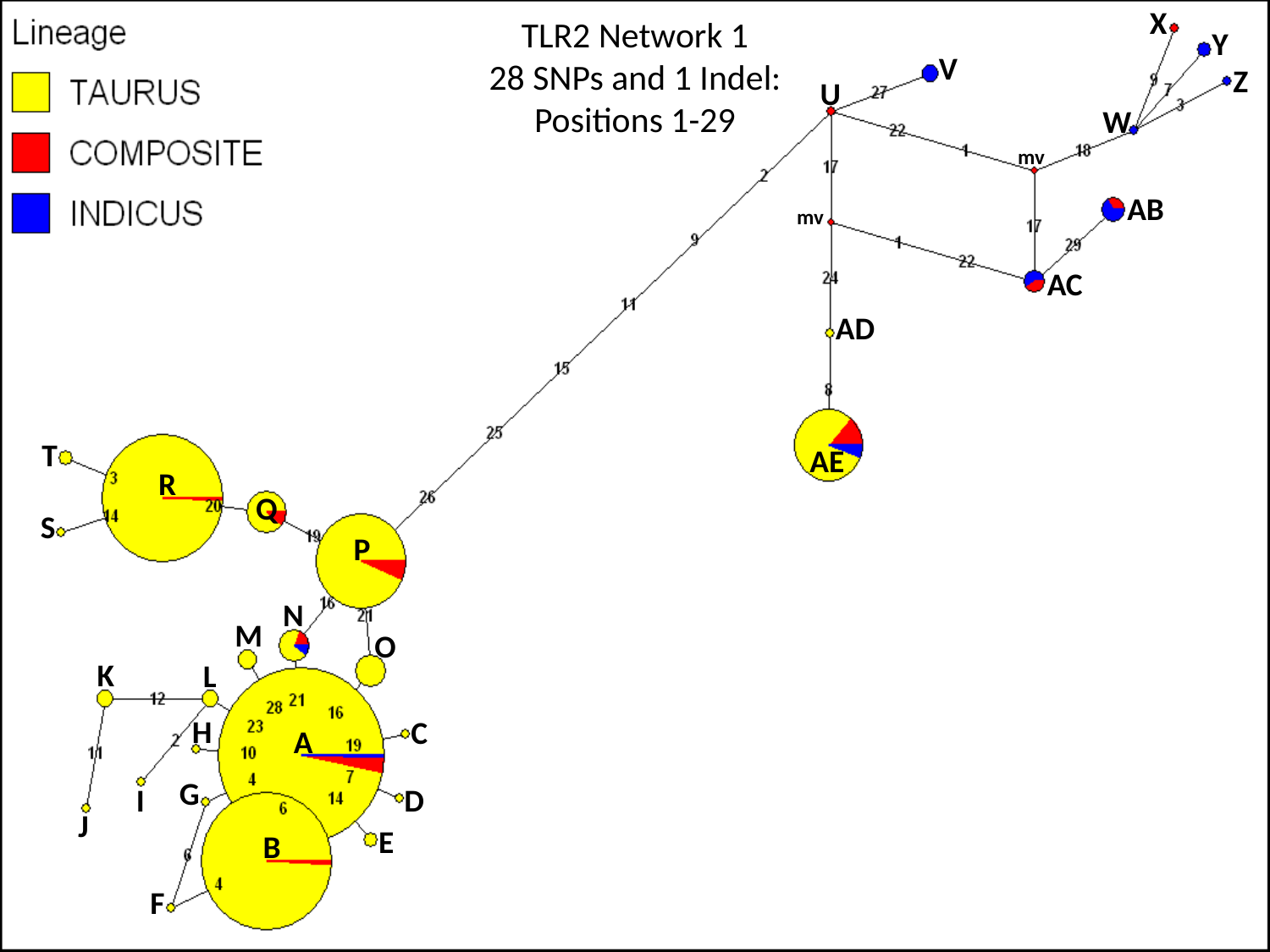

X
TLR2 Network 1
28 SNPs and 1 Indel: Positions 1-29
Y
V
Z
U
W
mv
AB
mv
AC
AD
T
AE
R
Q
S
P
N
M
O
K
L
H
C
A
G
I
D
J
E
B
F

## Slide 4
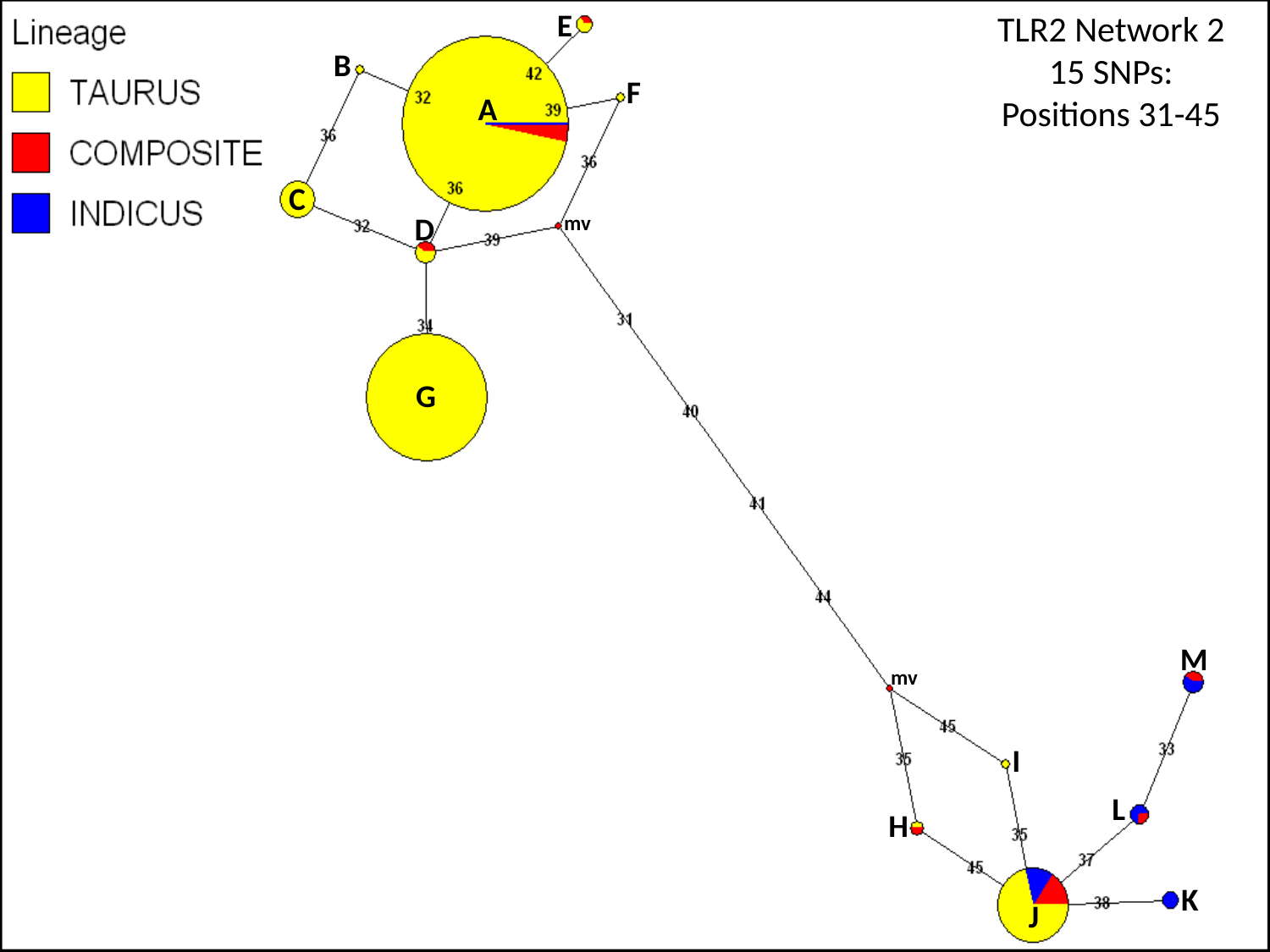

TLR2 Network 2
15 SNPs:
Positions 31-45
E
B
F
A
C
D
mv
G
M
mv
I
L
H
K
J

## Slide 5
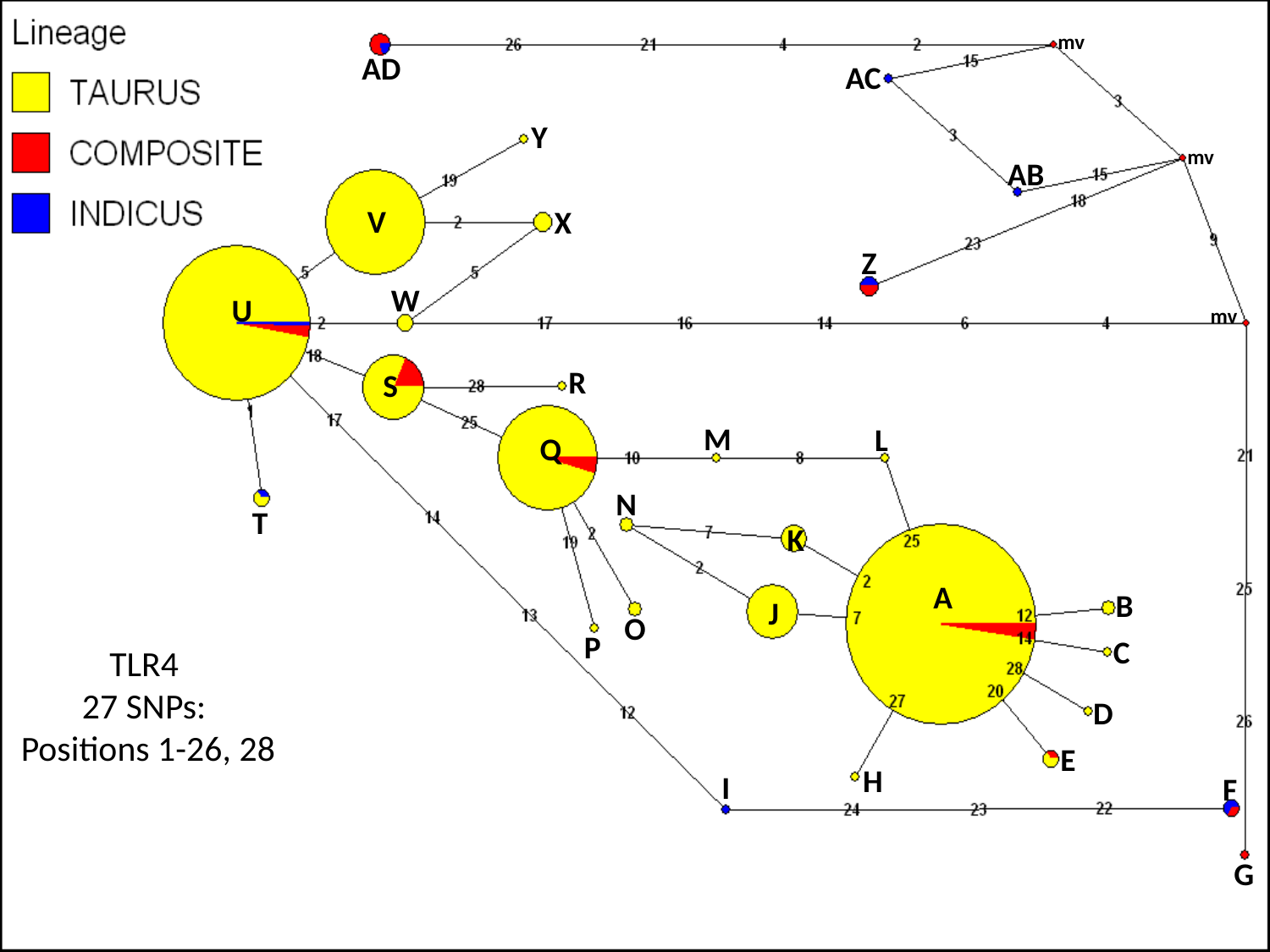

mv
AD
AC
Y
mv
AB
V
X
Z
W
U
mv
R
S
M
L
Q
N
T
K
A
B
J
O
P
C
TLR4
27 SNPs:
Positions 1-26, 28
D
E
H
I
F
G

## Slide 6
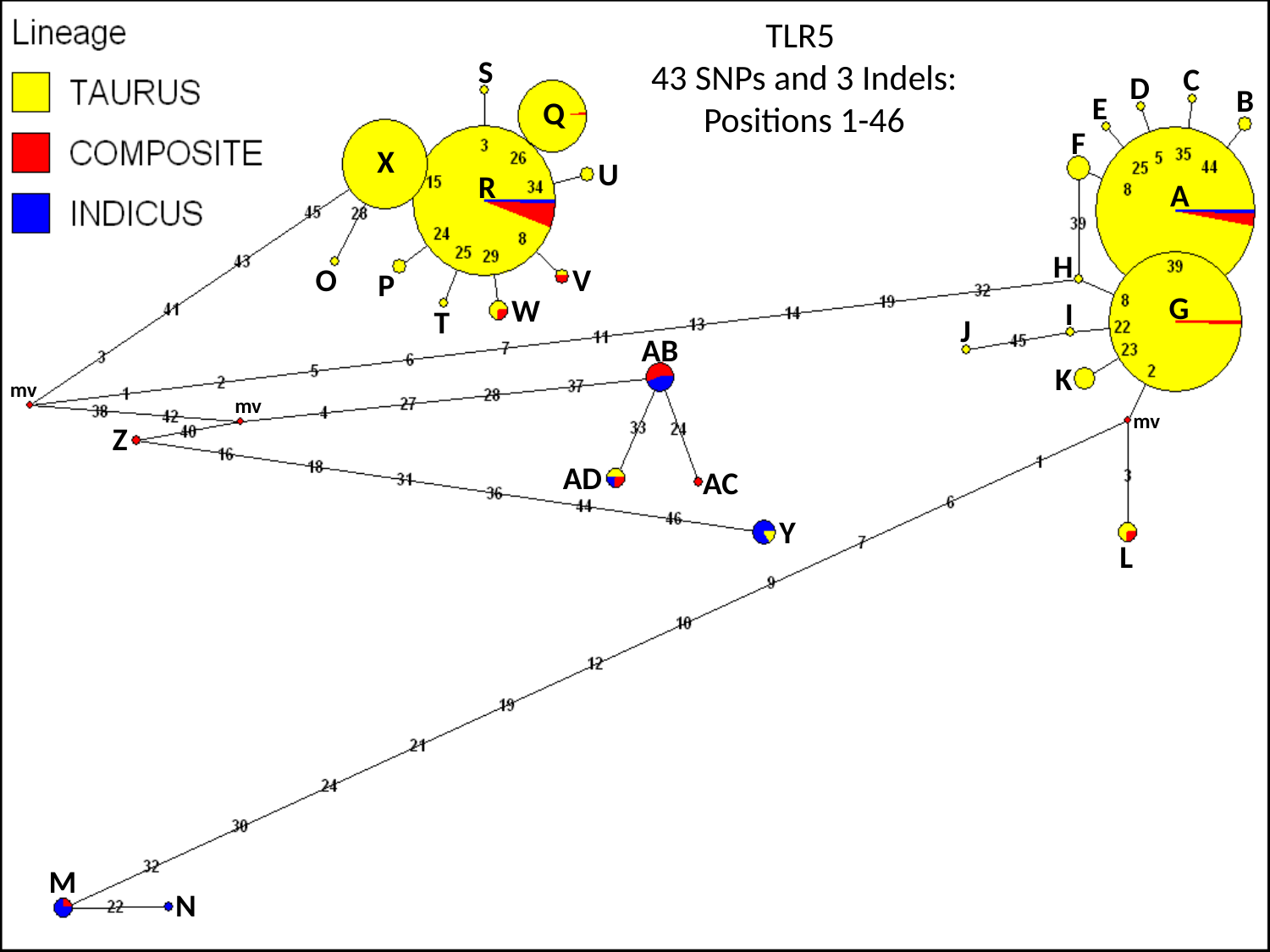

TLR5
43 SNPs and 3 Indels: Positions 1-46
S
C
D
B
E
Q
F
X
U
R
A
H
O
V
P
G
W
I
T
J
AB
K
mv
mv
mv
Z
AD
AC
Y
L
M
N

## Slide 7
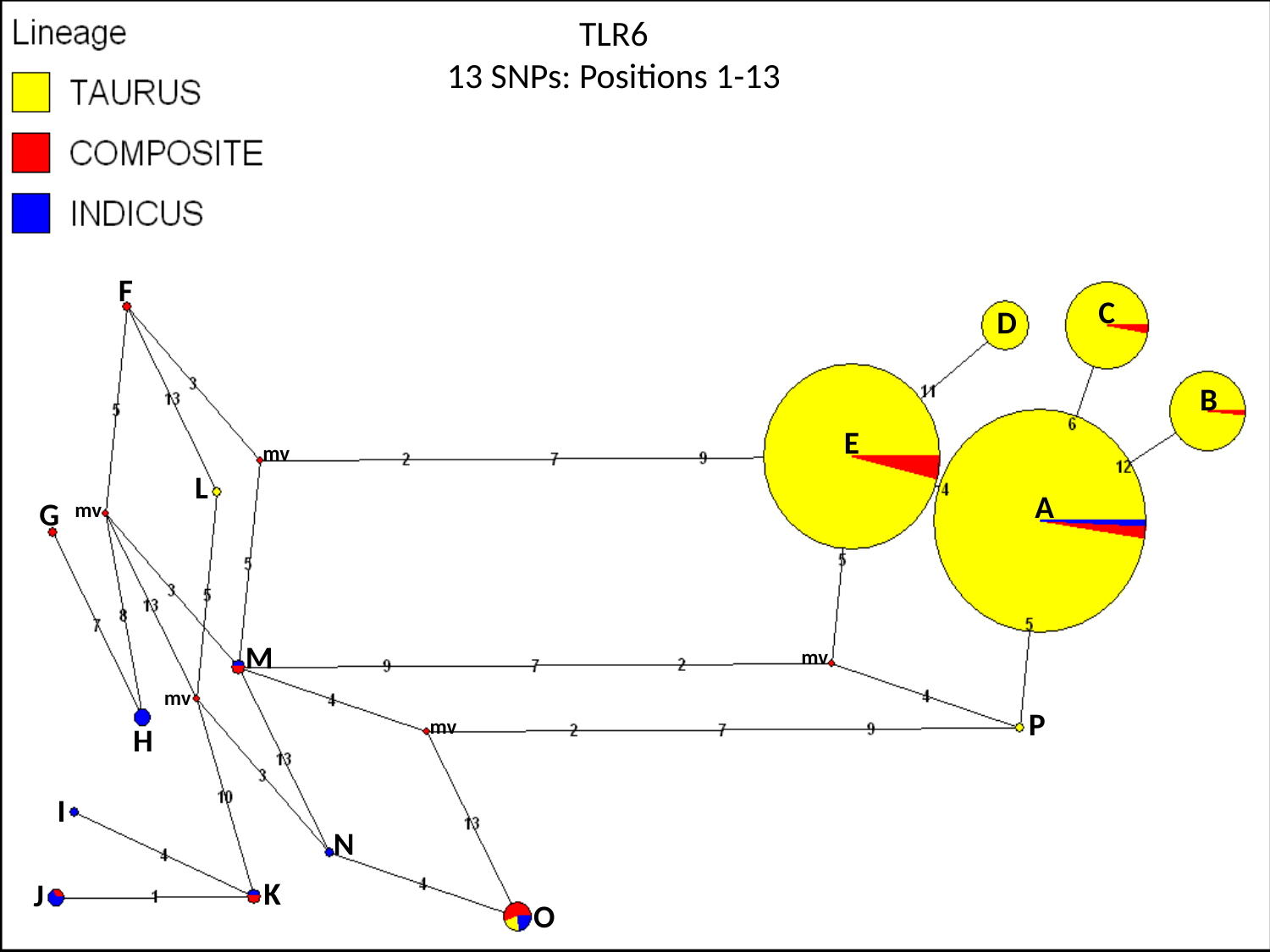

TLR6
13 SNPs: Positions 1-13
F
C
D
B
E
mv
L
A
G
mv
M
mv
mv
P
mv
H
I
N
K
J
O

## Slide 8
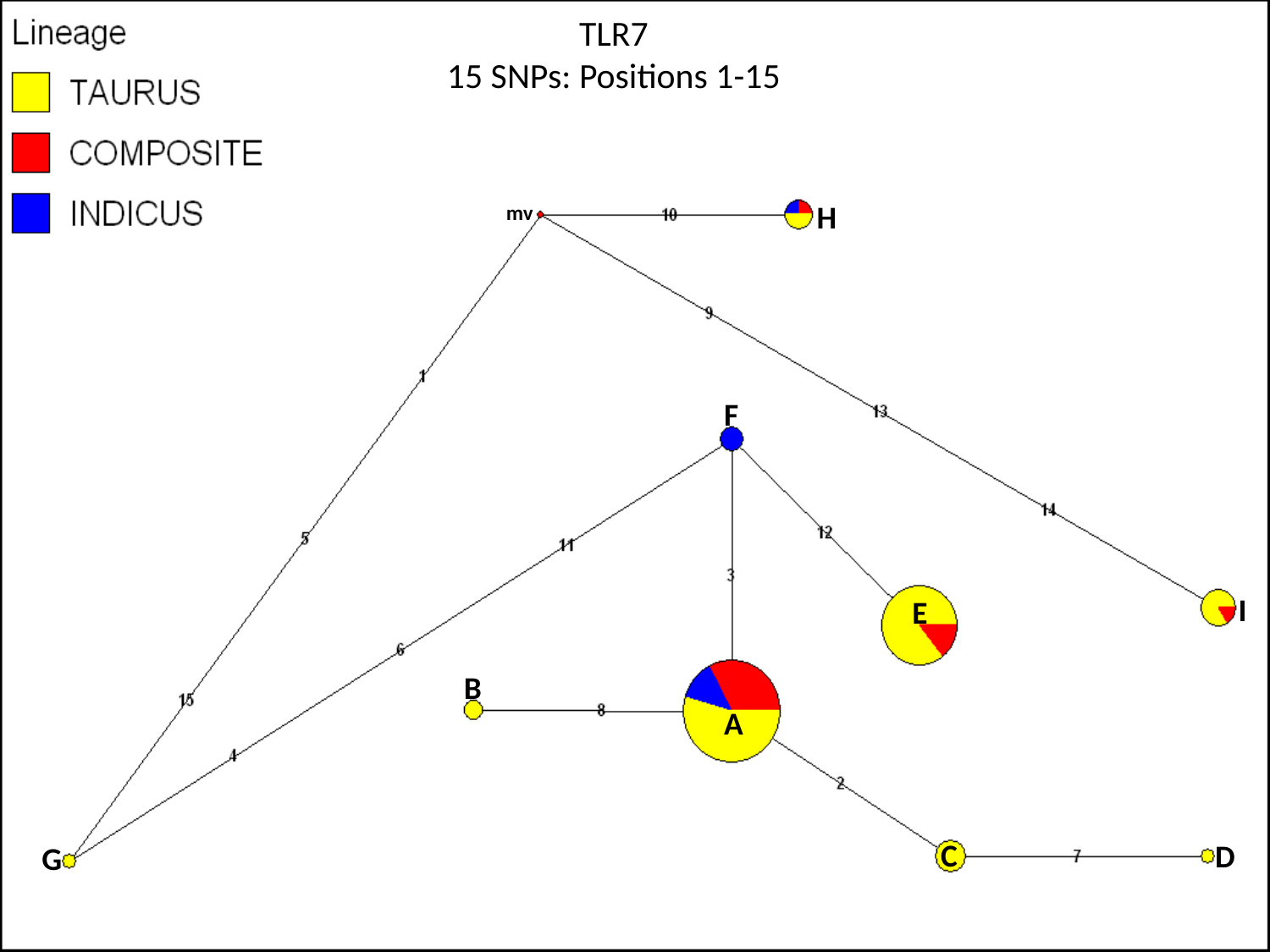

TLR7
15 SNPs: Positions 1-15
H
mv
F
I
E
B
A
C
D
G

## Slide 9
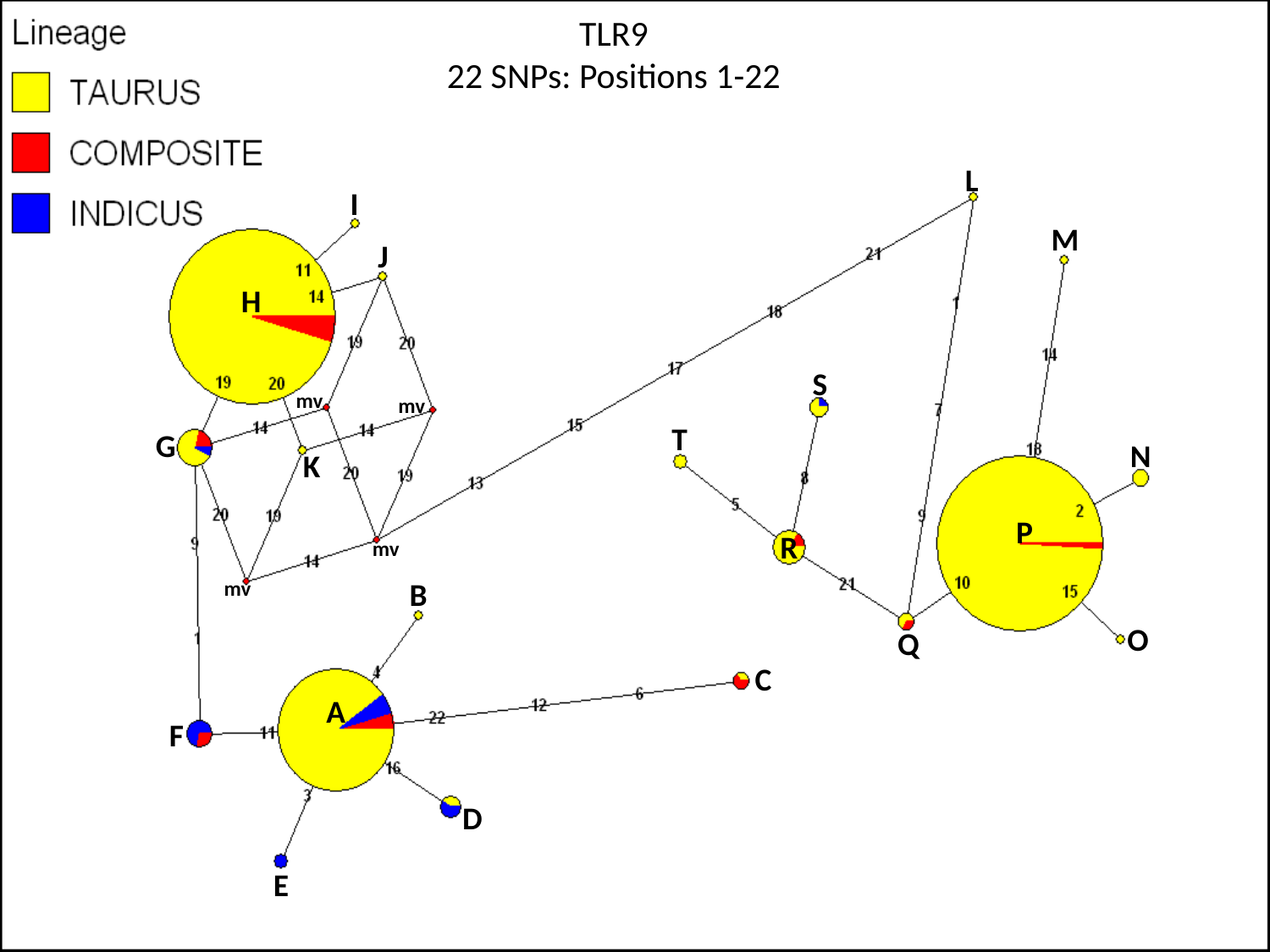

TLR9
22 SNPs: Positions 1-22
L
I
M
J
H
S
mv
mv
T
G
N
K
P
R
mv
B
mv
O
Q
C
A
F
D
E
